# Association of CSF proteins with tau and amyloid β levels in asymptomatic 70-year-olds

**DOI:** 10.1186/s13195-021-00789-5

**Published:** 2021-03-02

**Authors:** Julia Remnestål, Sofia Bergström, Jennie Olofsson, Evelina Sjöstedt, Mathias Uhlén, Kaj Blennow, Henrik Zetterberg, Anna Zettergren, Silke Kern, Ingmar Skoog, Peter Nilsson, Anna Månberg

**Affiliations:** 1 Division of Affinity Proteomics, Department of Protein Science, KTH Royal Institute of Technology, SciLifeLab, Tomtebodvägen 23A, Solna, Stockholm, Sweden; 2 Department of Neuroscience, Karolinska Institutet, Solna, Sweden; 3 Department of Psychiatry and Neurochemistry, Institute of Neuroscience and Physiology, The Sahlgrenska Academy, University of Gothenburg, Gothenburg, Sweden; 4 Clinical Neurochemistry Laboratory, Sahlgrenska University Hospital, Mölndal, Sweden; 5 Department of Neurodegenerative Disease, UCL Institute of Neurology, London, UK; 6 UK Dementia Research Institute at UCL, London, UK; 7 Neuropsychiatric Epidemiology Unit, Department of Psychiatry and Neurochemistry, Institute of Neuroscience and Physiology, Sahlgrenska Academy, Centre for Ageing and Health (AGECAP) at the University of Gothenburg, Gothenburg, Sweden; 8 Region Västra Götaland, Sahlgrenska University Hospital, Psychiatry, Cognition and Old Age Psychiatry Clinic, Gothenburg, Sweden

**Keywords:** Preclinical Alzheimer’s disease, Affinity proteomics, CSF markers, Brain-enriched proteins, Multidisciplinary epidemiological studies, AD pathophysiology

## Abstract

**Background:**

Increased knowledge of the evolution of molecular changes in neurodegenerative disorders such as Alzheimer’s disease (AD) is important for the understanding of disease pathophysiology and also crucial to be able to identify and validate disease biomarkers. While several biological changes that occur early in the disease development have already been recognized, the need for further characterization of the pathophysiological mechanisms behind AD still remains.

**Methods:**

In this study, we investigated cerebrospinal fluid (CSF) levels of 104 proteins in 307 asymptomatic 70-year-olds from the H70 Gothenburg Birth Cohort Studies using a multiplexed antibody- and bead-based technology.

**Results:**

The protein levels were first correlated with the core AD CSF biomarker concentrations of total tau, phospho-tau and amyloid beta (Aβ42) in all individuals. Sixty-three proteins showed significant correlations to either total tau, phospho-tau or Aβ42. Thereafter, individuals were divided based on CSF Aβ42/Aβ40 ratio and Clinical Dementia Rating (CDR) score to determine if early changes in pathology and cognition had an effect on the correlations. We compared the associations of the analysed proteins with CSF markers between groups and found 33 proteins displaying significantly different associations for amyloid-positive individuals and amyloid-negative individuals, as defined by the CSF Aβ42/Aβ40 ratio. No differences in the associations could be seen for individuals divided by CDR score.

**Conclusions:**

We identified a series of transmembrane proteins, proteins associated with or anchored to the plasma membrane, and proteins involved in or connected to synaptic vesicle transport to be associated with CSF biomarkers of amyloid and tau pathology in AD. Further studies are needed to explore these proteins’ role in AD pathophysiology.

**Supplementary Information:**

The online version contains supplementary material available at 10.1186/s13195-021-00789-5.

## Background

Understanding the evolution of molecular changes in neurodegenerative disorders such as Alzheimer’s disease (AD) is crucial for identification and validation of potential biomarkers for diagnostic, prognostic or therapeutic use. Apart from the hallmarks of AD, amyloid and tau pathology, several early changes related to AD have been identified, such as hypometabolism and structural changes within the brain [1]. Furthermore, large efforts are made to identify early changes in the cerebrospinal fluid (CSF) proteome [2–5], to complement measurements of amyloid beta (Aβ42), total tau (t-tau) and phospho-tau (p-tau). As one example, neurofilament light chain (NfL) has been recognized as a marker for neurodegeneration and could possibly be used to track progression of AD [6]. NfL has though been seen to increase in CSF in many neurodegenerative disorders apart from AD, such as frontotemporal dementia, Parkinson’s disease and amyotrophic lateral sclerosis [7]. Neurogranin (NRGN) and neuromodulin (GAP43) are two synaptic proteins also reported with a strong association with AD [8, 9], but in contrast to NfL, they seem to be more disease specific [2, 10–12]. Both proteins have shown altered levels before disease onset and correlate with tau CSF pathology [2, 12–14]. Synaptic loss and altered levels of synaptic proteins occur early in the disease course of AD and can be detected already in individuals with mild cognitive impairment (MCI) [15–17]. Neurodegeneration or neuronal death is not sufficient to explain the massive loss of synapses and it has been proposed that synapses are selectively removed before neuronal death [18]. A number of synaptic proteins apart from NRGN and GAP43 have been found altered before the onset of AD [19, 20]. In a cohort of older individuals with normal and impaired cognition, 22 proteins with altered levels in individuals with AD biomarker profiles, as defined by CSF levels of Aβ42, t-tau and p-tau, were identified [21]. Among the 790 proteins quantified, 59 were associated to Aβ42, t-tau or p-tau CSF pathology. Out of the 59, about half were classified as either brain-enriched or brain elevated according to the Human Protein Atlas.

In the present study, we investigated associations of 104 proteins, known to be brain-enriched or associated with different types of neurodegenerative disorders, with CSF biomarker evidence of AD pathology. The protein levels were explored in CSF from 307 asymptomatic 70-year-olds from the H70 Gothenburg Birth Cohort Studies (the H70 studies) [22, 23] using an affinity proteomic approach [2, 24–27]. The H70 studies are multidisciplinary epidemiological studies that examine birth cohorts representative of the older population in Gothenburg, Sweden. Levels of the 104 proteins were correlated to CSF concentration of Aβ42, t-tau and p-tau for all individuals together, as well as after dividing them into groups based on CSF Aβ42/Aβ40 ratio and Clinical Dementia Rating (CDR) score. The aim of the presented work was to explore how the levels of CSF proteins relate to Aβ42 and tau pathology, potentially shedding new light on the pathological processes preceding the development of AD.

## Material and methods

### Sample information

The samples included in the presented study were collected during the 2014–2016 examinations of the H70 Gothenburg Birth Cohort Studies in Gothenburg, Sweden. The individuals were obtained from the Swedish Population Registry and included both persons living in private households and in residential care. Every 70-year-old in Gothenburg, Sweden, born during 1944 on prespecified birthdates was invited to the examination in 2014–2016, and 1203 participated (response rate 72.2%) [22]. Of these, 430 (35.8%) consented to a lumbar puncture [23]. Contraindications (anticoagulant therapy, immune modulated therapy, cancer therapy) were present in 108, leaving 322 individuals suitable for inclusion (26.8%). CSF volume for the protein profiling analyses was insufficient for 10 participants, and five individuals with dementia were excluded, leaving 307 to be included in the presented analysis.

Briefly, 10 ml of CSF was collected into a polypropylene tube and centrifugated at 1800*g* for 10 min and thereafter stored at − 70 °C. Enzyme-linked immunosorbent assays (INNOTEST) were used to determine concentration of t-tau, p-tau and Aβ42. For NfL, an in-house sandwich enzyme-linked immunosorbent assay (ELISA) with capture and detection antibodies that were directed against the central rod domain of the protein (NfL 21 and NfL 23, respectively) was used [28]. An in-house ELISA method was also used for CSF NRGN [29]. For the CSF Aβ42/Aβ40 ratio, the V-PLEX Aβ Peptide Panel 1 (6E10) Kit (Meso Scale Discovery) was used. The method has been described in detail previously [22, 23].

All individuals included in the present study had undergone comprehensive neuropsychiatric and cognitive examinations and the prevalence of preclinical AD had been examined. Dementia was diagnosed according to the DSM-III-R criteria and used as an exclusion criterion. A more detailed description of the samples has been provided previously [23]. Blood samples were collected to establish genotyping for the single nucleotide polymorphisms (SNPs) rs7412 and rs429358 in *APOE* (gene map locus 19q13.2) using a KASPar® PCR SNP genotyping system (LGC Genomics) [22, 23]. Genotype data for these two SNPs were used to define ε2, ε3 and ε4 alleles. Out of the 307 individuals, *APOE* ε4 carrier status could be obtained for 304 individuals.

### Dichotomization of individuals

To explore if the associations to Aβ pathology change in the preclinical stages of AD, the individuals were divided into groups based on CSF Aβ42/Aβ40 ratio and CDR score. The cutoff-point for pathological Aβ42/Aβ40 ratio was 0.082, determined by the bimodal cut-point of data from the total sample with CSF measures on this variable (*n* = 318). Individuals with CSF Aβ42/Aβ40 < 0.082 were denoted as amyloid-positive (A+, *n* = 56) and individuals with CSF Aβ42/Aβ40 > 0.082 were denoted as amyloid-negative (A−, *n* = 251) (Table 1). For the assessment of cognitive function, individuals with a CDR score ≥ 0.5 were assigned to one group (*n* = 57) and individuals with a CDR score of 0 to another (*n* = 250) (Table 1). To further examine the effect of neurodegeneration and amyloid pathology on CSF protein levels, individuals were divided into four groups based on NfL concentration (median-based grouping) and CSF Aβ42/Aβ40 ratio combined (Supplementary Figure 1). The NfL groups were denoted Nf− for below median concentration and Nf+ for above median concentration and the subgroups were named as Nf−A− (*n* = 129), Nf−A+ (*n* = 24), Nf+A− (*n* = 122) and Nf+A+ (*n* = 32). In addition, individuals were divided into groups based on *APOE* ε4 carrier status and CSF Aβ42/Aβ40 ratio combined, denoted as APOEε4-A- (*n* = 179), APOEε4−A+ (*n* = 17), APOEε4+A− (*n* = 70) and APOEε4+A+ (*n* = 38) where APOEε4+ indicate carriers of the *APOE* ε4 allele (Supplementary Figure 1).

**Table 1:** Sample demographics

		Aβ42/Aβ40 ratio		CDR score	
	All	A+ < 0.082	A− > 0.082	≥ 0.5	0
***n***	307	56	251	57	250
**Sex** (F/M)	149/158	25/31	124/127	23/34	126/124
**Aβ42** (pg/ml)^a^	753 [145–1200]	396 [145–628]	792 [304–1200]	735 [195–1180]	761 [145–1200]
**Total tau** (pg/ml)^a^	299 [115–963]	412 [225–963]	287 [115–800]	303 [144–634]	298 [115–963]
**Phospho-tau** (pg/ml)^a^	48 [18–128]	59 [35–128]	45 [18–113]	49 [23–84]	47 [18–128]
**Aβ40** (pg/ml)^a^	6262 [2752–9968]	6618 [3661–9760]	6121 [2752–9968]	6042 [3175–8268]	6271 [2752–9968]
**Aβ42/Aβ40 ratio**	0.123 [0.022–0.210]	0.061 [0.022–0.081]	0.129 [0.083–0.210]	0.122 [0.034–0.199]	0.123 [0.022–0.210]
**NfL** (pg/ml)^a^	732 [255–12,312]	783 [302–3124]	711 [255–12,312]	720 [255–6233]	724 [276–12,312]
**Neurogranin** (pg/ml)^a^	197 [54–513]	224 [120–410]	188 [54–513]	199 [54–374]	196 [72–513]
***APOE*** **ε4 carrier** (Y/N/NA)	108/196/3	38/17/1	70/179/2	27/30/0	81/166/3
***APOE*** **genotype** (22/32/33/42/43/44/NA)	1/37/158/4/95/9/3	0/2/15/2/29/7/1	1/35/143/2/66/2/2	0/6/24/1/23/3/0	1/31/134/3/74/6/3
**CDR score** (0/0.5/1)	250/56/1	45/11/0	205/45/1	0/56/1	250/0/0

^a^Protein and peptide concentrations are presented in the format, median [range]

### Protein profiling

An antibody suspension bead array was used to explore the association between CSF markers (Aβ42, t-tau and p-tau) concentration and 104 proteins in human CSF. The selection of antibodies was based upon previously published and unpublished data generated within neurodegenerative disorders [2]. The majority of included antibodies were polyclonal rabbit IgG antibodies created within the Human Protein Atlas project (HPA, www.proteinatlas.org). Throughout the text and figures, proteins will be annotated by their HGNC ID.

The suspension bead array was created by immobilization of antibodies to magnetic colour coded carboxylated beads, as previously described [24, 30]. Fifteen microliters of each sample was diluted 1/2 into 96-well plates before labelling with a tenfold molar excess of biotin (21329, Thermo Scientific) as described before [24, 25]. Detection of captured proteins was enabled by addition of a streptavidin-coupled fluorophore (SA1004-4, Invitrogen). Read-out was done in a FlexMap3D instrument (Luminex Corporation), and results reported as median fluorescence intensities per bead identity and sample were calculated from at least 30 measured beads. More detailed explanations on experimental procedures can be found elsewhere [24, 31].

### Tissue expression and regional variation in the brain

To investigate the proteins included in this study further, we looked into the regional RNA expression profiles of the corresponding genes in human tissue, available in the HPA Tissue Atlas [32] and Brain Atlas [33]. The RNA tissue profiles are based on RNA expression from three datasets (HPA, FANTOM5 [34] and GTEx initiatives [35]) combined, to provide a complete expression overview of the human body. More details on the data normalization can be found elsewhere [36]. The normalized expression (NX) was downloaded from the HPA portal and plotted in a heatmap to visualize the RNA abundance across 37 tissues representing the whole body, as well as 12 major brain regions representing the brain.

### Immunohistochemical staining

Formalin-fixed paraffin-embedded tissue and antibodies produced within the HPA project were used for immunolabeling of selected targets. The experimental setup included both morphologically normal as well as AD brain tissue samples. The morphologically normal brain tissues were samples included in the HPA project pipeline (limited to cerebral cortex, caudate nucleus, hippocampus and cerebellum), which were anonymized and handled in accordance with Swedish laws and regulations (Uppsala Ethical Review Board reference # 2002-577, 2005-338 and 2007-159). The temporal cortex AD samples (AD1, male, age 83 and AD2, female, age 96) were obtained from the Netherlands Brain Bank (NBB), Netherlands Institute for Neuroscience (ethical permission no. EPN 2013/474-31/2). Tissues were cut in 4-μm-thin sections using Microm HM 355S water fall microtome (Thermo Fisher Scientific), placed on SuperFrost Plus glass slides (VWR) and baked at 40 °C overnight before the staining protocol was initiated by dewaxing in xylene, rehydration in graded alcohol including hydrogen peroxide. Heat-induced epitope retrieval was performed at 125 °C for 4 min in pressure boiler and pH 6.1 citrate buffer (S-169984-2, DAKO). The standard immunohistochemical protocol, with horseradish peroxidase polymer conjugated secondary antibody (TL-060-PH) and chromogenic 3,3′-diaminobenzidine (DAB, TA-002-QHCX) visualization, was used, in line with the previously described protocol [32]. The staining protocol was performed in an Autostainer 480 (Termo Fisher Scientific) with reagents from UltraVision™ Quanto Detection System HRP DAB-kit (Thermo Fisher Scientific) and primary antibody incubation for 30 min at room temperature. Antibody dilution factors were based on the previously optimized protocol with in the HPA-pipeline, the RPH3A (HPA002475) antibody was diluted 1:300, AMPH (HPA019828) diluted 1:1300 and TNR (HPA027150) diluted 1:60 in antibody diluent (TA-125-ADQ) prior to incubation.

### Data analysis and visualizations

All data was processed and visualized using the open source software R version 3.6.1 [37]. MA-Individual normalization [38] was applied to reduce potential plate differences, followed by adjustments based on position to minimize the effect of delay time during the detection step. Position adjustment was done by robust linear regression applied over sample plate position (*rlm* function, R package *MASS* version 7.3–51.4) [39]. Three identical wells of a sample pool were included in each assay plate to enable assessment of intra-assay reproducibility, and coefficients of variation (CV) were calculated for each antibody. The median CV across all antibodies was determined to 6.2% (IQR = 3.2). A subset of samples was experimentally re-analysed to asses inter-assay reproducibility and Lin’s concordance coefficient [40] was calculated to 0.985 (95% CI = 0.984–0.985) (*CCC* function, R package *DescTools* version 0.99.32) [41]. The overall correlation between assays was 0.97 (Spearman rho).

Two tailed *t* tests were used to determine differences in the concentration range of CSF markers (Aβ42, t-tau and p-tau) between groups. All correlations to CSF marker concentrations were calculated using Spearman’s rho statistics (*cor* and *cor.test* function, R package *stats*). Linear regression (*lm* function, R package *stats*) was performed to compare the association of the analysed proteins with CSF markers between groups: (1) A+ and A−, as defined by the Aβ42/Aβ40 ratio and (2) CDR ≥ 0.5 and CDR = 0. The linear regression models included log-transformed protein levels as the dependent variable, the interaction between the independent variables ‘CSF marker’ (Aβ42, t-tau or p-tau) and group, with sex and *APOE* ε4 carrier status as covariates. To explore if the associations with the CSF markers were affected by sex, additional linear regression models including protein levels as the dependent variable, the interaction between the independent variables ‘CSF marker’ (Aβ42, t-tau or p-tau) and sex, were performed. Wilcoxon rank sum tests (*wilcox.test* function, R package *stats*) were performed for analysis of protein level differences between A+ and A− individuals, and sex differences and differences between *APOE* ε4 carriers and non-carriers. Kruskal-Wallis tests were performed for analysis of differences between groups divided by NfL concentration, *APOE* ε4 carrier status and CSF Aβ42/Aβ40 ratio. Correlations to NRGN and NfL concentration were calculated using Pearson correlations (*cor* and *cor.test* function, R package *stats*). All NX values were log10-transformed before visualization and clustering of RNA tissue expression (*pheatmap* function, R package *pheatmap* version 1.0.12) [42]. To account for the parallel testing of all included analytes, all obtained *p* values were subjected to multiple testing corrections using Bonferroni correction (*p.adjust* function, R package *stats*) [43] and an adjusted *p* value below 0.05 was considered significant.

## Results

### Correlations with amyloid and tau pathology in all individuals

**Fig. 1:**
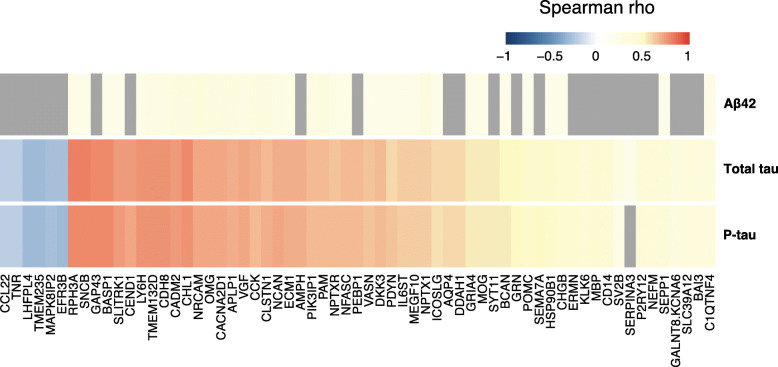
Protein correlation heatmap. A heatmap of all significant correlation coefficients for Aβ42, t-tau and p-tau based on protein levels from all individuals. Spearman rho values are indicated by the colour key. Non-significant correlations are presented in grey.

To determine how the analysed proteins relate to CSF concentrations of t-tau, p-tau and Aβ42, each protein was correlated with the three CSF markers. Significant associations with either t-tau, p-tau or Aβ42 were found for 63 proteins (Fig. 1, Supplementary Tables 1, 2 and 3). The strongest correlations with t-tau concentrations were identified for β-synuclein (SNCB) (Spearman rho = 0.80; *p* = 6E−69), rabphilin-3A (RPH3A) (Spearman rho = 0.80; *p* = 1E−67) and brain acid-soluble protein 1 (BASP1) (Spearman rho = 0.79; *p* = 8E−66). RPH3A and SNCB also displayed the strongest correlations to p-tau concentration together with neuromodulin (GAP43) (Spearman rho = 0.78; *p* = 1E−61). Neuronal cell adhesion molecule (NRCAM) showed the strongest correlation with Aβ42 concentration (Spearman rho = 0.33; *p* = 7E−07), followed by neuronal pentraxin-1 (NPTX1) (Spearman rho = 0.32; *p* = 1E−06) and voltage-dependent calcium channel subunit alpha-2/delta-1 (CACNA2D1) (Spearman rho = 0.31; *p* = 3E−06). Twenty-five proteins demonstrated significant correlations to t-tau and p-tau, but not Aβ42 (Fig. 1). Among these were GAP43, cell cycle exit and neuronal differentiation protein 1 (CEND1), amphiphysin (AMPH) and phosphatidyl-ethanolamine-binding protein 1 (PEBP1), all with moderate tau correlations (Spearman rho> 0.6). Although the majority of correlations were positive, we also observed weak negative correlations to both t-tau and p-tau concentrations for six proteins; transmembrane protein 235 (TMEM235), GABAA receptor regulatory, LHFPL tetraspan subfamily member 4 (LHFPL4), mitogen-activated protein kinase 8-interacting protein 2 (MAPK8IP2), protein EFR3 homolog B (EFR3B), tenascin-R (TNR) and C-C motif chemokine 22 (CCL22) (Fig. 1).

When comparing t-tau and p-tau concentration in all individuals, a strong positive correlation was observed between the two measurements (Spearman rho = 0.93; *p* = 1E−131, Supplementary Figure 2). The two tau concentrations did also show moderate correlations with Aβ40 concentration (Spearman rho> 0.77; *p* < 1E−59) but not Aβ42 concentration.

### Comparison of individuals grouped by CSF Aβ42/Aβ40 ratio

To explore if the associations with t-tau, p-tau and Aβ42 concentration change in the preclinical stage of AD, the individuals were dichotomized into two groups based on CSF Aβ42/Aβ40 ratio denoted as either amyloid-positive (A+) or amyloid-negative (A−) (Table 1). The ranges of t-tau, p-tau and Aβ42 CSF concentrations in both groups are illustrated in Supplementary Figure 3. Significant differences between the groups were found for all three markers (*p*_Aβ42_ = 2E−42, *p*_t-tau_ = 1E−07, *p*_p-tau_ = 1E−07). Statistically significant differences in protein levels between A+ individuals and A− individuals were identified for six proteins (Supplementary Figure 4). Among these were the previously mentioned proteins GAP43, SNCB, BASP1 and RPH3A, as well as dimethylarginine-dimethylaminohydrolase-1 (DDAH1) and aquaporin-4 (AQP4). All proteins displayed higher levels in the A+ individuals compared to the A− individuals.

### T-tau associations based on Aβ42/Aβ40 ratio

**Fig. 2:**
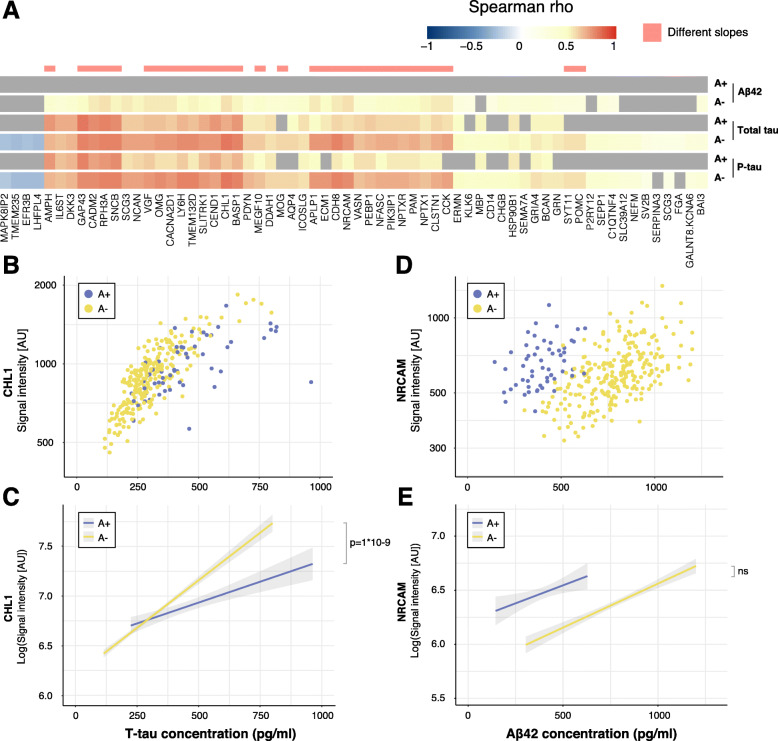
Associations with CSF markers for individuals divided by CSF Aβ42/Aβ40 ratio. **a** Heatmap of all significant correlation coefficients for individuals defined as A+ and A−. Spearman rho values are indicated by the colour key and grey colour represents non-significant correlations. Significant differences in slopes for association to t-tau and p-tau are indicated in pink. **b** Scatterplot of CHL1 levels and t-tau concentration. Both A+ and A− individuals display significant associations between CHL1 levels and t-tau concentration (A+: Spearman rho = 0.69; *p* = 5E−07, A−: Spearman rho = 0.80; *p* = 5E−56). **c** Linear regression revealed a significant difference between the slopes of CSF A+ and CSF A− individuals for the association between CHL1 and t-tau concentration (*t* = 7.13; *p* = 1E−09). **d** Scatterplot of NRCAM levels and Aβ42 concentration. A− individuals displayed a significant correlation between NRCAM levels and Aβ42 concentration (Spearman rho = 0.56, *p* = 4E−20) but not A+ individuals (Spearman rho = 0.36, *p* = 0.8). **e** Linear regression showed no significant difference between slopes of A+ and A− individuals for the association between NRCAM and Aβ42 concentration

The majority of the 63 proteins with significant correlations with t-tau in all individuals remained significant in both the A− (61/63) and A+ (41/63) groups (Fig. 2a, Supplementary Table 1). Yet, 33 proteins demonstrated significant differences in slopes between the two groups using linear regression models (Fig. 2a, Table 2, Supplementary Table 1). Neural cell adhesion molecule L1-like protein (CHL1) displayed the largest association to CSF t-tau concentration in the A− individuals (Spearman rho = 0.80; *p* = 5E−56), as well as a significant difference in slopes (*t* = 7.13; *p* = 1E−09; *R*^2^ = 0.64, Fig. 2b, c) while GAP43 showed the largest association in the A+ group (Spearman rho = 0.82; *p* = 1E−12; *t* = 5.80; *p* = 2E−06; *R*^2^ = 0.66). However, the most significant slope differences were seen for transmembrane protein 132D (TMEM132D) (*t* = 7.88; *p* = 8E−12; *R*^2^ = 0.64) and lymphocyte antigen 6H (LY6H) (*t* = 7.26; *p* = 4E−10; *R*^2^ = 0.63). Significant differences in the slopes were not found for any of the proteins with a negative association to t-tau.

**Table 2:** Proteins displaying significant slope differences between A+ and A− individuals sorted by t-tau rho

				Aβ42 concentration		T-tau concentration			P-tau concentration		
Protein name	HGNC ID	Uniprot ID	Antibody	rho	***p***	rho	***p***	***p*** (slope)	rho	***p***	***p*** (slope)
*β-synuclein*	SNCB	Q16143	HPA035876	0.20	4E−02	0.80	6E−69	2E−05	0.79	8E−65	ns
*Rabphilin-3A*	RPH3A	Q9Y2J0	HPA002475	0.26	4E−04	0.80	1E−67	4E−07	0.79	4E−65	4E−03
*Brain acid-soluble protein 1*	BASP1	P80723	HPA050333	0.21	2E−02	0.79	8E−66	2E−08	0.78	1E−61	5E−04
*Neuromodulin*	GAP43	P17677	PA5–34943	ns	ns	0.79	5E−65	2E−06	0.78	1E−61	9E−03
*Neural cell adhesion molecule L1-like protein*	CHL1	O00533	HPA003345	0.30	7E−06	0.77	2E−60	1E−09	0.77	1E−58	1E−05
*Cadherin-8*	CDH8	P55286	HPA014908	0.28	5E−05	0.75	1E−55	3E−09	0.75	7E−54	2E−05
*Lymphocyte antigen 6H*	LY6H	O94772	HPA077218	0.25	8E−04	0.75	2E−54	4E−10	0.75	4E−54	2E−06
*Transmembrane protein 132D*	TMEM132D	Q14C87	HPA010739	0.28	1E−04	0.75	5E−54	8E−12	0.75	6E−54	3E−08
*Cell cycle exit and neuronal differentiation protein 1*	CEND1	Q8N111	HPA042527	ns	ns	0.74	2E−53	4E−06	0.72	2E−48	8E−03
*SLIT and NTRK like family member 1*	SLITRK1	Q96PX8	HPA012414	0.21	2E−02	0.74	7E−53	2E−08	0.73	3E−50	2E−04
*Cell adhesion molecule 2*	CADM2	Q8N3J6	HPA010024	0.31	5E−06	0.74	6E−52	1E−04	0.73	1E−50	4E−02
*Oligodendrocyte-myelin glycoprotein*	OMG	P23515	HPA008206	0.31	4E−06	0.71	2E−46	5E−06	0.72	2E−48	9E−04
*Voltage-dependent calcium channel subunit alpha-2/delta-1*	CACNA2D1	P54289	HPA008213	0.31	3E−06	0.71	5E−46	9E−07	0.71	3E−47	5E−04
*Neurosecretory protein VGF*	VGF	O15240	HPA055177	0.29	3E−05	0.71	2E−45	1E−03	0.71	3E−47	ns
*Neuronal cell adhesion molecule*	NRCAM	Q92823	HPA061433	0.33	7E−07	0.70	1E−44	3E−08	0.72	2E−47	6E−06
*Neurocan core protein*	NCAN	O14594	HPA058000	0.28	1E−04	0.69	5E−43	6E−06	0.70	1E−44	3E−03
*Amyloid-like protein 1*	APLP1	P51693	HPA028971	0.31	6E−06	0.69	1E−42	6E−07	0.69	2E−43	1E−04
*Extracellular matrix protein 1*	ECM1	Q16610	HPA027241	0.27	3E−04	0.69	4E−42	4E−08	0.68	2E−41	2E−05
*Amphiphysin*	AMPH	P49418	HPA019828	ns	ns	0.69	6E−42	3E−03	0.68	2E−41	ns
*Cholecystokinin*	CCK	P06307	HPA069515	0.30	1E−05	0.67	7E−40	5E−06	0.66	9E−38	5E−04
*Calsyntenin-1*	CLSTN1	O94985	HPA012749	0.31	5E−06	0.67	3E−39	2E−04	0.67	2E−39	2E−02
*Neurofascin*	NFASC	O94856	HPA073444	0.24	2E−03	0.66	4E−38	4E−06	0.66	3E−38	2E−04
*Phosphoinositide-3-kinase-interacting protein 1*	PIK3IP1	Q96FE7	HPA002959	0.28	8E−05	0.66	4E−37	9E−07	0.65	1E−35	5E−04
*Peptidyl-glycine alpha-amidating monooxygenase*	PAM	P19021	HPA042260	0.30	1E−05	0.66	4E−37	2E−04	0.65	8E−36	4E−02
*Phosphatidyl-ethanolamine-binding protein 1*	PEBP1	P30086	HPA063904	ns	ns	0.65	9E−37	3E−03	0.67	8E−39	ns
*Dickkopf-related protein 3*	DKK3	Q9UBP4	HPA011164	0.21	2E−02	0.65	2E−36	1E−02	0.66	3E−37	ns
*Neuronal pentraxin receptor*	NPTXR	O95502	HPA001079	0.27	3E−04	0.64	2E−35	3E−03	0.67	5E−39	4E−02
*Vasorin*	VASN	Q6EMK4	HPA011246	0.21	2E−02	0.64	2E−34	5E−04	0.64	1E−34	ns
*Neuronal pentraxin-1*	NPTX1	Q15818	HPA077062	0.32	1E−06	0.61	2E−30	2E−02	0.61	8E−31	ns
*Multiple epidermal growth factor-like domains protein 10*	MEGF10	Q96KG7	HPA026876	0.24	3E-03	0.60	1E−29	6E−03	0.61	9E−31	ns
*Myelin oligodendrocyte glycoprotein*	MOG	Q16653	AMAb91067	0.23	6E−03	0.54	1E−22	2E−04	0.54	4E−22	1E−02
*Synaptotagmin-11*	SYT11	Q9BT88	HPA064091	ns	ns	0.54	2E−22	2E−02	0.54	4E−22	ns
*Pro-opiomelanocortin*	POMC	P01189	HPA063644	0.22	2E−02	0.49	4E−18	2E−04	0.49	7E−18	7E−03

### P-tau associations based on Aβ42/Aβ40 ratio

Similarly as for t-tau, the majority of significant associations to p-tau in all individuals were not affected by dividing the cohort based on CSF Aβ42/Aβ40 ratio (Fig. 2a and Supplementary Table 2). In the A− individuals 60/62 associations remained significant and 35/62 in the A+ group. The linear regression models revealed significant differences in slopes for 24 proteins, all significant for t-tau as well (Table 2). Again, the most significant slope differences were seen for TMEM132D (*t* = 6.57; *p* = 3E−08; *R*^2^ = 0.58) and LY6H (*t* = 5.58; *p* = 2E−06; *R*^2^ = 0.58), which also displayed significant associations to p-tau concentration in both A+ and A− individuals.

### Aβ42 associations based on Aβ42/Aβ40 ratio

When dividing individuals into A+ and A−, 50 proteins displayed significant associations to Aβ42 concentration in the A− individuals. However, no significant associations were found in the A+ group (Fig. 2a and Supplementary Table 3). Again, NRCAM was the protein with the largest association to Aβ42 concentration, although only in the A− individuals (Spearman rho = 0.56; *p* = 4E−20, Fig. 2d). Linear regression models showed no significant difference in slopes between A+ and A− individuals for NRCAM (Fig. 2e) or any other protein although a few models could explain up to 30% of the variation in protein levels (Supplementary Table 3).

### Comparison of individuals grouped by CDR score

To determine if there were any differences in associations with CSF t-tau, p-tau or Aβ42 concentrations based on CDR scores, individuals were divided into two groups: CDR ≥ 0.5 (*n* = 57) and CDR = 0 (*n* = 250) (Table 1). The range of t-tau, p-tau and Aβ42 concentrations in both groups are illustrated in Supplementary Figure 3. No significant differences between the groups were found for either CSF marker. There were also no significant differences in protein levels observed between individuals with CDR = 0 and CDR ≥ 0.5 (data not shown).

### T-tau associations based on CDR score

A total of 60 proteins displayed a significant association to t-tau in individuals with CDR = 0, all of which did also display significant associations in the A− group. Thirty-one proteins showed significant associations to t-tau concentration in the CDR ≥ 0.5 group. GAP43 showed the most significant association in individuals with CDR ≥ 0.5 (Spearman rho = 0.72, *p* = 3E−08, Supplementary Figure 5A, Supplementary Table 4) but no significant difference in slopes between individuals with CDR ≥ 0.5 and CDR = 0 (Supplementary Figure 5B). When dividing individuals into groups based on CDR score, none of the studied proteins obtained significant differences in their slopes using linear regression.

### P-tau associations based on CDR score

The same 60 proteins which showed significant associations to t-tau concentration in the CDR = 0 group also had a significant association to p-tau concentration (Supplementary Table 5). In the CDR ≥ 0.5 group, 34 proteins presented significant associations to p-tau concentration. The majority (30/34) of these were the same as for t-tau associations and GAP43 was again the protein with strongest association (Spearman rho = 0.75, *p* = 2E−09). Significant differences in the slopes were not found for any of the proteins after linear regression.

### Aβ42 associations based on CDR score

Dividing individuals based on CDR score revealed 18 proteins with significant associations to Aβ42 concentration (Supplementary Table 6) in individuals with CDR = 0, all of which were also significant when dividing the individuals into A+ and A−. NRCAM also showed a significant association to Aβ42 concentration in the CDR ≥ 0.5 individuals (Spearman rho = 0.47, *p* = 3E−02). Linear regression modeling resulted in no proteins with significant difference in slopes between CDR ≥ 0.5 and CDR = 0 individuals.

### Sex differences

Linear regression did reveal a significant contribution of sex for two of the 63 previously mentioned proteins, TNR and CCL22. Furthermore, levels of regulating synaptic membrane exocytosis protein 3 (RIMS3), vascular cell adhesion protein 1 (VCAM1) and chitinase-3-like protein 1 (CHI3L1), von Willebrand factor C domain-containing protein 2-like (VWC2L) and C-type lectin domain family 2 member L (CLEC2L) did also show a significant contribution of sex in the linear regression models (Supplementary Table 7). Wilcoxon rank sum tests did display significant differences on a group level between females and males for RIMS3 and VCAM-1 only (Supplementary Table 7 and Supplementary Figure 6). Further examination showed that, although different between groups, sex had no significant interaction with the CSF markers for any of the seven proteins (Supplementary Table 7).

### NfL, NRGN and *APOE* ε4 carrier status

**Fig. 3:**
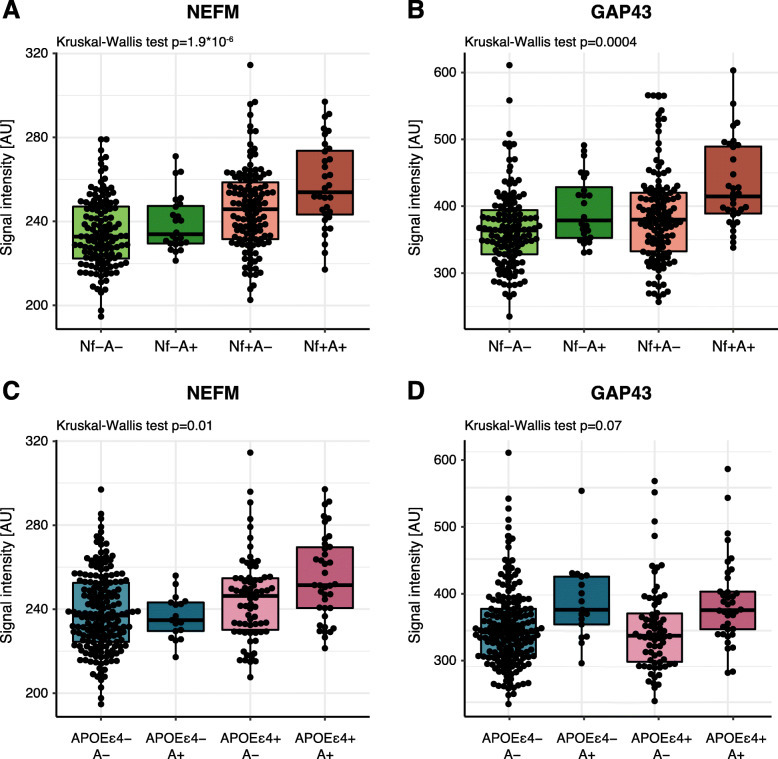
NEFM and GAP43 levels after stratification based on CSF Aβ42/Aβ40, NfL concentration and *APOE* ε4 carrier status. **a** Visualization of NEFM levels in individuals divided by NfL concentration and CSF Aβ42/Aβ40 ratio. **b** Visualization of GAP43 levels in individuals divided by NfL concentration and CSF Aβ42/Aβ40 ratio. Higher levels of GAP43 were identified in Nf+A+ individuals. **c** Visualization of NEFM levels in individuals divided by *APOE* ε4 carrier status and CSF Aβ42/Aβ40 ratio. *APOE* ε4 carriers displayed higher levels of NEFM. **d** Visualization of GAP43 levels in individuals divided by *APOE* ε4 carrier status and CSF Aβ42/Aβ40 ratio. A+ individuals displayed higher levels of GAP43 although the trend was not statistically significant

The obtained protein profiles were furthermore investigated in relation to NfL and NRGN, two of the suggested markers of neurodegeneration and synaptic dysfunction, as well as *APOE* ε4 carrier status. The measured NfL concentrations did not display strong correlations to any of the other suggested markers for AD, neurodegeneration or synaptic dysfunction (rho < 0.3, Supplementary Figure 2). Weak significant correlations to NfL concentration were found for neurofilament medium chain (NEFM), glutamine synthetase (GLUL) and glutamate decarboxylase 1 (GAD1) (Pearson *R* = 0.23–0.30; *p* <  0.01) (data not shown). No significant differences could be observed when comparing NfL concentration between A+ and A− individuals. However, upon stratifying individuals into groups based on both CSF Aβ42/Aβ40 ratio and NfL concentration, nine proteins displayed significant differences between groups (Fig. 3 and Supplementary Figure 7). Two trends could be identified among the nine proteins: (i) higher protein levels in Nf+ individuals, independently of Aβ42/Aβ40 ratio and (ii) higher protein levels in the Nf+A+ group. The NEFM protein was found at higher levels in Nf+ individuals (Fig. 3a) whereas GAP43 mainly displayed higher levels in the Nf+A+ individuals (Fig. 3b). Nonetheless, the dichotomization did not contribute to any new trends in the interaction between group and the CSF markers (data not shown).

The measured NRGN concentration showed moderate to strong correlations with Aβ40, t-tau and p-tau concentration (Supplementary Figure 2). Correlating the 104 analysed protein profiles to NRGN concentration revealed 71 proteins with significant associations, of which 37 displayed a Pearson *R* >  0.5 (Supplementary Figure 8A). Significantly higher NRGN concentrations were identified in the A+ individuals compared to the A− individuals (*p* = 3E−06, Supplementary Figure 8B).

NEFM was the only protein that displayed a significant difference in protein levels between *APOE* ε4 carriers and non-carriers (*p* < 0.01, data not shown), a difference that was observed again when combining *APOE* ε4 status and CSF Aβ42/Aβ40 ratio to divide individuals into groups. NEFM levels were higher in APOEε4+ individuals independently of CSF Aβ42/Aβ40 ratio (Fig. 3c). GAP43 instead showed a trend of higher levels in A+ individuals regardless of *APOE* ε4 carrier status, although the difference was not statistically significant (Fig. 3d). Dichotomization of individuals based on CSF Aβ42/Aβ40 ratio and *APOE* ε4 status did not affect the interaction between group and the CSF markers (data not shown).

### Tissue expression and regional variation in the brain

To characterize the proteins included in this study, we looked further into the variation in RNA expression of the corresponding genes in human tissues, using tissue expression profiles available in the Human Protein Atlas (HPA) [32]. When comparing the expression levels across 37 different human tissues, the genes separated into four clusters. One cluster with more general expression in all tissue types (Cluster 1), one with elevated expression in the liver compared to other tissues (Cluster 2), one with higher expression in the brain (Cluster 3) and one last mixed group with high expression in brain or other tissue types (Cluster 4) (Supplementary Figure 9). The majority of the studied proteins were found in the cluster with high expression in the brain and lower expression in the remaining tissues (Cluster 3). Cluster 2 contained none of the proteins for which significant correlations were observed.

**Fig. 4:**
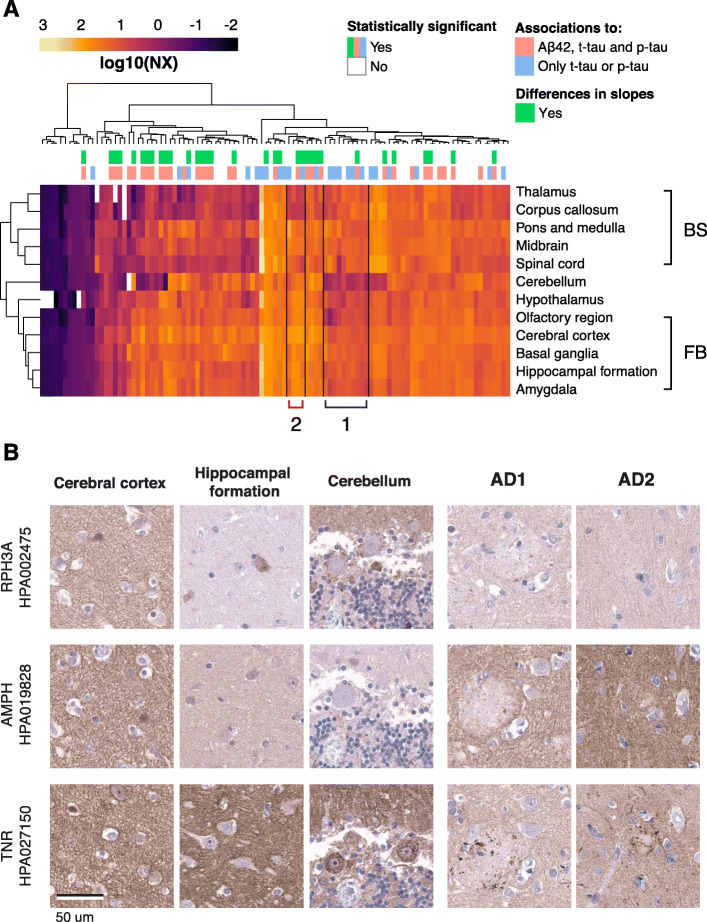
Regional brain expression and tissue expression of representative proteins. **a** Clustering of RNA expression data showed brain region clustering of the forebrain and brainstem. Two clusters identified visually included genes with lower expression in the cerebellum compared to other brain regions and higher expression in brainstem regions compared to forebrain (Cluster 1). A few genes with lower expression in white matter-rich regions such and thalamus could also be identified (Cluster 2). **b** Distribution of RPH3A, AMPH and TNR in cerebral cortex, hippocampal formation and cerebellum from a healthy donor, as well as temporal cortex from two AD patients. The three selected proteins displayed a similar staining pattern in cerebral cortex as well as a general neuropil positivity in the AD tissue. Furthermore, TNR showed positive staining around the amyloid plaques

Gene expression analysis based on regional brain profiles (HPA Brain Atlas) [33] showed that the gene equivalent of most proteins included in this study were expressed throughout the human brain. The only examples of genes that were classified as regionally elevated, defined as a 4-fold higher expression level in one region compared to all other regions, were pro-opiomelanocortin (POMC) (hypothalamus), potassium voltage-gated channel subfamily C member 1 (KCNC1, cerebellum), proenkephalin-B (PDYN, basal ganglia) and cholecystokinin (CCK, forebrain regions). Clustering of gene expression showed brain region clustering of the forebrain versus brainstem (Fig. 4a). This analysis also visually identified included genes with a lower expression in the cerebellum compared to other brain regions and slightly higher expression in brainstem regions compared to forebrain (Cluster 1). Additionally, a few genes with lower expression in the white matter-rich regions corpus callosum and thalamus could also be identified in a cluster (Cluster 2) and included neuronal markers such as NEFM, microtubule-associated protein 2 (MAP2), BASP1 and GAP43. In summary, very few genes could be classified as regionally elevated within the brain and no clear trends or clustering based on correlations to the core AD biomarkers were found. However, Cluster 1 contained a large proportion of the genes for which the corresponding proteins correlated to tau- but not Aβ42 levels.

No clear differences regarding protein location or cellular specificity were observed when comparing the available spatial protein information in IHC images available at the Human Protein Atlas. Both neuronal, glial and neuropil staining patterns were identified and the patterns were seemingly independent of the observed correlations to Aβ42, t-tau or p-tau. Even so, three representative proteins with different association profiles (RPH3A, AMPH and TNR) were selected for additional immunohistochemical staining in normal brain tissue as well as samples from AD patients in order to confirm their spatial distributions and investigate potential disease-associated differences (Fig. 4b). RPH3A displayed strong correlations to tau concentration, significant slope differences between A+ and A− individuals, and a weak correlation to Aβ42 concentration. AMPH did also show strong correlations to tau concentration but no correlation to Aβ42 concentration. TNR was one of the few proteins displaying a weak negative correlation to tau concentration. All three selected proteins displayed a similar staining pattern in the cerebral cortex and hippocampal formation, detected in subsets of neuronal cell bodies and with general neuropil positivity. However, the detailed location in cerebellum differed between the three proteins. RPH3A demonstrated positivity of interneurons in the molecular layer as well as neuropil positivity. AMPH showed positivity in neuropil and synaptic connections in the granular layer, while TNR mainly showed neuropil positivity. The proteins displayed a similar general neuropil positivity in the AD tissue, but TNR also showed positivity associated to the plaques which AMPH and RPH3A did not.

## Discussion

In this study, we analysed 104 proteins in CSF from asymptomatic 70-year-olds and examined their relationship to the core AD CSF biomarkers Aβ42, p-tau and t-tau, reflecting amyloid pathology, tau pathology and neurodegeneration, respectively. When correlating levels in all included individuals about half of the analysed proteins showed significant associations with at least one of these AD biomarkers. The Aβ42/Aβ40 ratio was then used to divide individuals into two groups, with A+ individuals potentially representing the preclinical stage of AD, and A− individuals representing healthy ageing. The majority of proteins remained significantly correlated to t-tau and p-tau in both sample groups, but correlations to Aβ42 were only found in the A− group. A total of 33 proteins showed significant differences in the slopes of the two groups when examining their relationship to t-tau, using linear regression.

Most of the proteins with significantly different slopes in relation to CSF tau levels did also display a weaker significant association to CSF Aβ42 levels, such as CHL1 and NRCAM (rho_t-tau_ > 0.7, rho_Aβ42_ > 0.3). Interestingly, the proteins GAP43, CEND1, AMPH and synaptotagmin-11 (SYT11) did not. Especially noteworthy are GAP43 and CEND1, both displaying strong correlations (rho > 0.7) to t-tau and p-tau, but no association to Aβ42. Accumulation of aggregated Aβ42 into plaques and p-tau into tangles occurs alongside each other in the development of AD [44], and we hypothesize that proteins differently associated with them also reflect different biological events. Two protein groups could be identified among the 33 proteins with significantly different slopes. The first group consists of transmembrane proteins, proteins associated to or anchored to the plasma membrane, and the second group contains proteins involved in or connected to synaptic vesicle transport. The recognition molecules CHL1, NRCAM and NFASC that are all part of the L1 family were among the proteins with strong correlations to tau levels, and differential correlations between A+ and A− individuals. These transmembrane proteins are critical during neuronal development, as they influence axonal outgrowth, neuronal migration, synapse formation and synaptic plasticity [45–47]. CHL1 is processed and cleaved by BACE1 [48], the major beta secretase giving rise to amyloid β peptides in AD through cleavage of the amyloid precursor protein (APP). It has been found at lower levels in CSF from MCI patients as well as patients with AD [49]. Furthermore, NRCAM has been shown to be cleaved by ADAM10, which acts as an alpha-secretase for APP [50]. Similarly to CHL1, NRCAM has also been found at lower levels in CSF from patients with AD [51, 52]. Knock-out of ADAM10 did also affect the processing of CHL1 and SLIT and NTRK like family member 1 (SLITRK1), suggesting that they too are cleaved by ADAM10. NFASC has been identified at higher levels in CSF from MCI patients compared to controls using mass spectrometry [20]. In the same study, this was also observed for neurocan core protein (NCAN) which is known to interact with cell adhesion molecules such as proteins in the L1 family. When stratifying the MCI group into stable MCI and MCI-AD, as defined by altered CSF biomarker levels, the authors observed that MCI-AD individuals displayed higher levels of both NFASC and NCAN. The classical cadherins is another protein family of transmembrane proteins that are calcium dependent and believed to work as synaptic recognition molecules. Experimental evidence point towards their importance for synaptic formation and specificity [53]. We found cadherin-8 (CDH8) to correlate strongly to t-tau and p-tau in all individuals. Previously, CDH8 has been found enriched in glutamatergic synapses of cortical neurons in mice [54] and polymorphisms in the *CDH8* gene has been implicated in autism [55]. Cell adhesion molecule 2 (CADM2) has been found to affect axon guidance [56] and belongs to the SynCAM family that, similarly to the L1 family and the cadherins, mediates cell-cell adhesion and is enriched at the synaptic terminals [47]. Neuronal pentraxin receptor (NPTXR) is another synaptic receptor protein that has been implicated in AD together with several of the proteins mentioned above [57, 58]. The transmembrane protein TMEM132D is highly expressed in the brain [59] and has been proposed to serve as an oligodendric cell surface marker [60] with cell adhesion functions [61], although its main function still remains unknown. To our knowledge, TMEM132D has not been studied in the context of AD but we previously observed altered levels in patients with frontotemporal dementia [31]. Other membrane-bound proteins that showed strong associations to tau were CEND1, myelin oligodendrocyte glycoprotein (MOG), PIK3IP1 and VASN, which have various functions relevant to cell cycle regulation, cell adhesion and T cell activation [62–65].

Apart from transmembrane proteins, many of the proteins with strong correlations to t-tau and p-tau CSF levels are synaptic proteins associated to or anchored to the membrane such as BASP1, GAP43 and CACNA2D1. BASP1 and GAP43 are both calcium-binding growth-associated proteins, often implicated in similar pathways [66–68]. GAP43 is mainly located to the presynaptic terminals and plays a crucial role in neuronal development by modulating the assembly of actin during axonal growth and has also been shown to affect synaptic plasticity [69–71]. As previously mentioned, higher levels of GAP43 has been observed in CSF from patients with AD and individuals in the preclinical stages of AD, similarly to what has been seen for NRGN [2, 12]. GAP43 and BASP1 have previously showed significant associations with tau concentration, together with for example the cancer-associated protein LY6H, and PEBP1 which is believed to have implications for AD [21, 72, 73]. The *CACNA2D1* gene encodes the protein α_2_δ-1 which is a subunit of voltage-gated calcium channels present in skeletal muscle. In the brain and spinal cord, it is located at the presynaptic terminals and more specifically in the presynaptic boutons [74]. Disruptions in the *CACNA2D1* gene have been linked to epilepsy and intellectual disability in a few individuals [75]. CADM2, CHL1, CDH8, NFASC, NPTXR and SLITRK1 have also been seen to localize to the synapses, as stated above [47, 54, 58, 76, 77].

The second group of proteins found to have strong correlations to t-tau and p-tau, included SNCB, AMPH, RPH3A and SYT11, all involved in synaptic vesicle transport. α-synuclein has long been connected to the etiology of Parkinson’s disease [78] but the normal function of the synucleins remains to be elucidated. A previous study using an αβγ-Syn^−/−^ mouse model could show that all three synucleins (α, β and γ) are important for synaptic vesicle endocytosis [79]. They have all been identified at elevated levels in AD CSF [80] but SNCB could be of particular interest as the altered levels are seen in AD but not frontotemporal dementia, Parkinson’s disease nor amyotrophic lateral sclerosis [81]. The BAR-domain family protein AMPH is highly abundant throughout the nervous system and is also involved in synaptic vesicle endocytosis [82, 83]. AMPH2 has been implicated in AD [84] but AMPH has to our knowledge, not been studied in the context of AD. However, decreasing levels of AMPH have been identified in a tauopathy mouse model [85], possibly reflecting mechanisms relevant to AD. RPH3A and SYT11 are two calcium-dependent proteins likewise involved in synaptic vesicle transport [86–88].

An obvious topic for discussion is whether the observed correlations between the abovementioned proteins and CSF tau levels are the result of early neurodegeneration or basal secretion of neuronal proteins to interstitial fluid and CSF. As mentioned before, synaptic dysfunction is believed to occur prior to the formation of both amyloid plaques and neurofibrillary tangles, implying that soluble Aβ and tau could disturb synaptic function [89–91]. Aβ oligomers are believed to affect synaptic function by increasing glutamate levels and inducing overactivation of NMDA receptors [18, 92]. A variety of other synaptic receptors are also directly affected by high Aβ levels as they have been shown to interact with Aβ oligomers. The activation of such receptors can in turn induce an inflammatory response disrupting the synapses. Soluble tau has also been observed to interact with proteins present at the synapses, like cell adhesion molecules [93] and intracellular proteins [94]. Both Aβ and phosphorylated tau are observed at synaptic terminals in mouse models of AD and post mortem brains from patients with AD [95]. Aβ and tau levels also correlate with reduction in the number of synapses [96] and in mouse models overexpressing human tau, tau levels correlate with a decrease in synaptic proteins [18, 90]. There is, however, also a physiological activity-dependent neuronal release of tau and Aβ to the extracellular space [97, 98]. Additionally, neuronal tau secretion may be stimulated by Aβ pathology [99] and consequently, Aβ stimulated secretion of tau might be expected to occur in A+ individuals. If Aβ also induces secretion of additional proteins, this would potentially explain the correlations reported here but it remains to be further examined.

We observed strong correlations between tau levels and a number of synaptic proteins in CSF. The abovementioned studies were all done on brain tissue and most synaptic proteins measured in CSF have been found to increase in the preclinical stages of AD and MCI [2, 19, 20, 100]. It has been hypothesized that the high levels of synaptic proteins observed in CSF from the MCI-AD group could be the result of a compensatory mechanism to the loss of synapses, as was identified in the early 1990s [20, 101, 102]. It has further been proposed that the concentration of the same proteins decline in the later stages of disease, as a result of additional synaptic loss. Most of the proteins presented in our study were found to correlate positively with t-tau and p-tau levels independently of CSF Aβ42 levels, although with differences in the slope, i.e. the protein:tau ratio. Generally, we observed a lower incline for the A+ group compared to the A− group. It appears as both groups follow the same trajectory until a certain point, after which the slope for the A+ group flattens out. If this has anything to do with the fact that most of these individuals are positive not only for Aβ42, but t-tau or p-tau as well, will have to be investigated further. One could speculate that there might be disease processes already initiated in the individuals with high t-tau and p-tau, processes such as synaptic degradation or compensatory mechanisms. These events could alter the protein levels, resulting in a loss of the relation of tau and protein level that is observed in a healthy state. It should however be noted that the majority of individuals studied here are cognitively healthy and therefore different from patients with MCI or prodromal AD. Though some of these individuals might develop AD in the future, this is likely many years from the time of sampling. It remains to be seen from future sample collections if and how their CSF protein profiles will change.

Basal secretion from neurons to the extracellular matrix and CSF may to some extent explain why the proteins measured in our study correlate to CSF levels of t-tau, p-tau and Aβ42. This is especially true for the intracellular proteins involved in synaptic vesicle transport or the proteins anchored to plasma membrane. The antibodies used for detection of the transmembrane proteins in our study target epitopes predicted to be on the outside of the plasma membrane but the method does not allow us to determine in what format the proteins are detected. In previous reports, membrane proteins were among the largest protein class detected in CSF [103, 104]. Further studies will elucidate whether we are measuring the result of actively secreted cleavage products or full-length proteins leaking during cellular degradation.

In order to confidently use a biological fluid such as CSF as a way of exploring disease pathogenesis, it is important to identify its normal between-individual variation. Several efforts to establish the normal CSF protein concentration range has been initiated throughout the years [105–108]. The inter-individual differences in the CSF proteome are generally thought to be larger than the intra-individual differences [106]. However, many proteins display low variation between individuals and extracellular proteins have been found to exhibit lower variation compared to intracellular proteins [108]. The correlations we see between CSF tau and a number of the proteins analysed in our study could very well be a reflection of individual differences in CSF dynamics, where certain individuals have higher levels of all brain-derived proteins, including tau, in their CSF compared to others. Because we observe many proteins correlating strongly to tau concentration, many of the analysed proteins do also correlate strongly with each other. If these correlations are a result of tau-related processes or a reflection of normal physiological events cannot be answered by the data presented here. The same is true when it comes to stratifying the individuals based on the Aβ42/Aβ40 ratio. All individuals included in our study were recruited through the H70 Gothenburg Birth Cohort study and the majority are cognitively healthy. Those with a CDR score > 0 displayed very mild symptoms and all were regarded as asymptomatic, independent of CSF Aβ and tau levels. Though it is possible that the individuals with pathological amyloid levels represent a very early stage in the development of AD, it is also possible that some of them have inherently low CSF protein levels, also affecting the observed amyloid levels. The only way to learn more about the between-individual variation in the CSF proteome and how it affects protein biomarkers is by continuing to explore the protein profiles of both healthy and diseased individuals.

Upon stratification of individuals into subgroups, a few proteins displayed significantly higher levels in the A+ individuals compared to A− individuals. These differences likely reflect the correlation to tau levels, although DDAH1 and AQP4 were not among the proteins with the strongest correlations to tau. Additional stratification based on both CSF Aβ42/Aβ40 ratio and NfL concentration revealed two trends in protein levels. Higher levels of proteins in the Nf+ individuals, independently of Aβ42/Aβ40 ratio, could reflect processes related to non-AD specific neurodegeneration and were observed for two structural proteins, NEFM and MBP, as well as the inflammatory protein SERPINA3. The Nf+A+ individuals show patterns of a broader neurodegeneration and could thus be expected to show an altered protein profile compared to the other individuals. Stratification based on CSF Aβ42/Aβ40 ratio and *APOE* ε4 carrier status did only display a significant trend in the levels of NEFM, with higher levels in the *APOE* ε4 carriers independently of CSF Aβ42/Aβ40 ratio. To our knowledge, there are no published studies investigating the effect of *APOE* ε4 carrier status on NEFM levels but neither CSF nor plasma NfL concentration seem to be significantly affected by *APOE* ε4 carrier status [109, 110].

Using linear regression, we did not observe any significant differences in slopes between the CDR ≥ 0.5 and CDR = 0 groups. This lack of difference is not surprising when relating our findings to the classical model of AD biomarkers [1]. Differences in the molecular markers such as CSF Aβ42, CSF tau and PET-amyloid burden can be detected many years before the onset of symptoms. However, the CDR score might not be sensitive enough to detect a meaningful difference between a patient with a score of 0 and a patient with a score of 0.5 on a molecular level. Something that is illustrated by the lack of difference between the two groups with regard to the core CSF AD biomarkers.

Whereas we did not observe sex to have a confounding effect on the proteins most strongly correlated to the CSF core biomarkers, we did find effects on the association of a few proteins, such as RIMS3, VCAM1 and CHI3L1. All these have also previously been observed to differ between the sexes on a genetic or proteomic level [111–113].

The gene expression data revealed four distinct clusters when compared across 37 different human tissue types. The majority of proteins with significant correlations to any of the three core AD biomarkers were found in the third cluster, displaying high expression in brain compared to the other tissues. As the selection of proteins was partly based on genes classified as brain-enriched, the observed pattern was to be expected. Despite this, it is interesting to note that no proteins with high liver expression was among those with associations to the AD markers since they are likely to be mostly derived from the blood. When clustering gene expression data from the twelve main brain regions, an overall grouping of the forebrain region versus the brain stem regions was observed. This is in concordance with the previously published regional clustering based on global gene expression [33]. One gene cluster with lower expression in the cerebellum and slightly higher expression in the brain stem regions compared to the forebrain was observed. Several of these genes were oligodendrocyte-associated and abundant in the white matter of the brain. The neuronal proteins GAP43, BASP1 and NEFM with low expression in white matter-rich regions were also seen to cluster together. BASP1 and GAP43 are often implicated in similar pathways, as mentioned previously, and it is perhaps not surprising that they display similar gene expression profiles as well. Apart from the expected enrichment of genes highly expressed in the brain, no clear trends could be seen related to the correlations found to Aβ42, t-tau or p-tau.

When comparing immunohistochemical stainings of three representative synaptic proteins, RPH3A, AMPH and TNR, we observed similar distribution patterns across the control cerebral cortex and hippocampal formation as well as the AD tissue samples. RPH3A was among the proteins with the strongest correlation to both t-tau and p-tau concentration and did also display a weak correlation to Aβ42 concentration. Furthermore, the A+ and A− individuals showed significantly different associations between RPH3A levels and both t-tau and p-tau concentration. AMPH was also among the proteins with a strong correlation to t-tau and p-tau concentration, but in contrast to RPH3A, it did not demonstrate any correlation to Aβ42 concentration. Moreover, the group differences in association to p-tau did not reach statistical significance. TNR was among the few proteins that presented a weak negative correlation to t-tau and p-tau concentrations and interestingly the only of the three examples with a distinct immunolabeling associated to the plaques observed in the AD brain samples. To conclude, we could not identify any clear trends or relationships between the association to AD CSF biomarker concentration and protein distribution patterns in the brain areas studied here.

### Limitations

There are several limitations to the presented study. The study was cross-sectional, which limits possibilities to draw conclusions regarding the direction of associations. Although the H70 CSF sample was relatively large for a population-based cohort, the number of samples in the different subgroups was small yielding limitations in the statistical power. The cut-offs used for dichotomization of individuals into groups might not be optimal, especially in regard to the division by NfL concentration for which we used the median concentration. Furthermore, all individuals included in the study are healthy and cognitively unimpaired; hence, identifying large differences between these groups could perhaps not be expected. Follow-up information about the cognitive status of the included individuals would have been highly valuable for the interpretation of our results; however, at the time of writing, this information is not available. The absence of a validation cohort limits the interpretation of the results as some of the identified trends might be specific to this cohort and non-generalizable to a broader population. Lastly, these findings are of observational character and mechanistic conclusions cannot be drawn from the presented data.

## Conclusion

In summary, we identified a large number of proteins significantly correlated to the core AD CSF biomarkers in asymptomatic 70-year-olds. When dividing individuals into groups based on CSF Aβ42/Aβ40 ratio, a few proteins also displayed significantly different slopes between the two groups, albeit with similar trends. While the correlations we have identified are highly interesting, we do not intend to conclude on any causal relationship between these proteins and disease pathology [114]. As discussed above, differences in basal secretion related to neurodegeneration or differences in CSF dynamics are two factors that could contribute to the observed pattern, and it is possible that several of these proteins are reflecting different biological events that occur in parallel. In order to understand if the proteins studied here are involved in or affected by tau and amyloid pathology present in AD, their relationship would have to be explored in functional studies. To increase the understanding of how this potential relationship change over time and with disease progression, longitudinal samples as well as clinical follow-up information is needed. This information as well as new samples will be collected from the H70 participants over time and become the subject for evaluation within future studies.

## Supplementary Information

**Additional file 1: Supplementary Table 1.** Rho values and linear regression results for total-tau correlations in all individuals and with individuals divided by Aβ42/Aβ40 ratio. **Supplementary Table 2.** Rho values and linear regression results for phospho-tau correlations in all individuals and with individuals divided by Aβ42/Aβ40 ratio. **Supplementary Table 3.** Rho values and linear regression results for Aβ42 correlations in all individuals and with individuals divided by Aβ42/Aβ40 ratio. **Supplementary Table 4.** Rho values and linear regression results for total-tau correlations in all individuals and with individuals divided by CDR score. **Supplementary Table 5.** Rho values and linear regression results for phospho-tau correlations in all individuals and with individuals divided by CDR score. **Supplementary Table 6.** Rho values and linear regression results for Aβ42 correlations in all individuals and with individuals divided by CDR score. **Supplementary Table 7.** *P*-values for linear regressions and Wilcoxon rank sum tests regarding sex differences.

**Additional file 2: Supplementary Figure 1.** Dichotomization of individuals into groups based on NfL concentration and *APOE* ε4 carrier status.

**Additional file 3: Supplementary Figure 2.** Correlation scatterplots between suggested markers for AD, neurodegeneration or synaptic dysfunction. All correlations with a Spearman rho value> 0.6 are displayed in red.

**Additional file 4: Supplementary Figure 3.** Range of CSF markers in individuals divided by CSF Aβ42/Aβ40 ratio or CDR score. Significant differences are indicated with stars, *** *p* < 0.001.

**Additional file 5: Supplementary Figure 4.** Boxplots of proteins displaying significant differences between A+ individuals and A- individuals.

**Additional file 6: Supplementary Figure 5.** T-tau association with GAP43 levels for individuals divided by CDR score. (A) Scatterplot of GAP43 levels and t-tau concentration. Both individuals with CDR ≥ 0.5 and CDR = 0 display significant associations of GAP43 levels with t-tau concentration (CDR ≥ 0.5: Spearman rho = 0.72; *p* = 3E-08; CDR = 0: Spearman rho = 0.81; *p* = 4E-56). (B) Linear regression revealed no significant difference between slopes of CDR ≥ 0.5 and CDR = 0 individuals for the association of GAP43 with t-tau concentration.

**Additional file 7: Supplementary Figure 6.** Sex differences for RIMS3 and VCAM1. Significant differences between female and male protein levels could be identified using the Wilcoxon rank sum test. However, sex showed no significant interaction with the CSF markers.

**Additional file 8: Supplementary Figure 7.** Boxplots of proteins displaying significant differences between individuals divided by NfL concentration and CSF Aβ42/Aβ40. Upon stratification of individuals based on both CSF Aβ42/Aβ40 ratio and NfL concentration two trends in protein profiles could be identified; higher protein levels in Nf + individuals, independently of Aβ42/Aβ40 ratio and higher protein levels in the Nf + A+ group.

**Additional file 9: Supplementary Figure 8.** Correlations to NRGN concentration and comparison of NRGN levels between A+ and A- individuals. (A) Visualization of Pearson R for correlations between NRGN concentration and the 104 measured proteins. Thirty-seven proteins displayed a Pearson R > 0.5 and are annotated by their HGNC ID. (B) Higher concentration of NRGN was observed in A+ individuals compared to the A- individuals.

**Additional file 10: Supplementary Figure 9.** Heatmap of tissue expression for all studied proteins across 37 human tissues. When comparing the expression profiles of the 104 analysed proteins across 37 different human tissues, the responding genes could be divided into four clusters. The first cluster showed a general expression in all tissue types (Cluster 1) and the second cluster displayed elevated expression in the liver compared to other tissues (Cluster 2). A third cluster had higher expression in the brain (Cluster 3) and the last was a mixed group with high expression in brain or other tissue types (Cluster 4).

## Data Availability

Anonymised data will be shared upon request using the Science for Life laboratories Data Repository and the file will be available using the DOI: 10.17044/scilifelab.13280867 after publication.

## Abbreviations

Aβ40: Amyloid beta, the 40 amino acid form
Aβ42: Amyloid beta, the 42 amino acid form
AD: Alzheimer’s disease
AMPH: Amphiphysin
APOE: Apolipoprotein E
APP: Amyloid precursor protein
BASP: Brain acid-soluble protein 1
CACNA2D1: Voltage-dependent calcium channel subunit alpha-2/delta-1
CADM2: Cell adhesion molecule 2
CCK: Cholecystokinin
CCL22: C-C motif chemokine 22
CDR: Clinical Dementia Rating
CEND1: Cell cycle exit and neuronal differentiation protein 1
CHI3L1: Chitinase-3-like protein 1
CHL1: Neural cell adhesion molecule L1-like protein
CLEC2L: C-type lectin domain family 2 member L
CSF: Cerebrospinal fluid
DDAH1: Dimethylarginine-dimethylaminohydrolase-1
EFR3B: Protein EFR3 homolog B
GAD1: Glutamate decarboxylase 1
GAP43: Neuromodulin
GLUL: Glutamine synthetase
HPA: Human Protein Atlas
KCNC1: Potassium voltage-gated channel subfamily C member 1
LHFPL4: LHFPL tetraspan subfamily member 4
LY6H: Lymphocyte antigen 6H
MAP2: Microtubule-associated protein 2
MAPK8IP2: Mitogen-activated protein kinase 8-interacting protein 2
MCI: Mild cognitive impairment
MOG: Myelin oligodendrocyte glycoprotein
NCAN: Neurocan core protein
NEFM: Neurofilament medium chain
NfL: Neurofilament light chain
NFASC: Neurofascin
NPTX1: Neuronal pentraxin-1
NPTXR: Neuronal pentraxin receptor
NRCAM: Neuronal cell adhesion molecule
NRGN: Neurogranin
NX: Normalized expression
P-tau: Phosphorylated tau
PDYN: Proenkephalin-B
PEBP1: Phosphatidyl-ethanolamine-binding protein 1
PIK3IP1: Phosphoinositide-3-kinase-interacting protein 1
POMC: Pro-opiomelanocortin
RPH3A: Rabphilin-3A
SLITRK1: SLIT and NTRK like family member 1
SNCB: β-synuclein
SNPs: Single nucleotide polymorphisms
SYT11: Synaptotagmin-11
T-tau: Total tau
The H70 studies: The H70 Gothenburg Birth Cohort Studies
TMEM132D: Transmembrane protein 132D
TMEM235: Transmembrane protein 235
TNR: Tenascin-R
VASN: Vasorin
VCAM1: Vascular cell adhesion protein 1
VWC2L: Willebrand factor C domain-containing protein 2-like
