# Supplementary figures and images for: Association of CSF proteins with tau and amyloid β levels in asymptomatic 70-year-olds
Source: Alzheimers Res Ther. 2021 Mar 2;13:54. doi: 10.1186/s13195-021-00789-5 (PMC7923505; doi:10.1186/s13195-021-00789-5)

Nf-L concentration and A $\beta$ 42/A $\beta$ 40 ratio

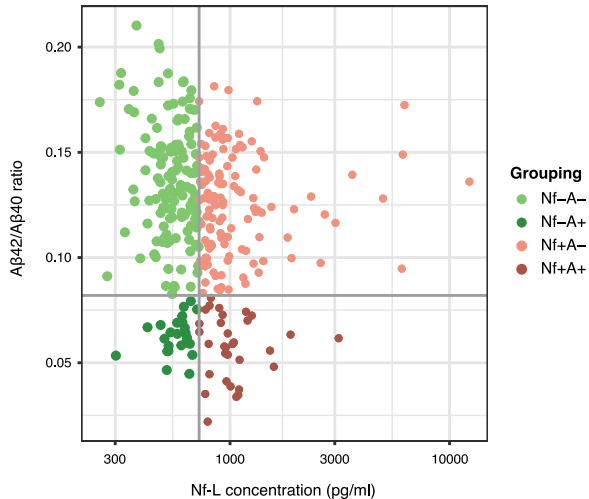

APOE $\epsilon$ 4 carrier status and A $\beta$ 42/A $\beta$ 40 ratio

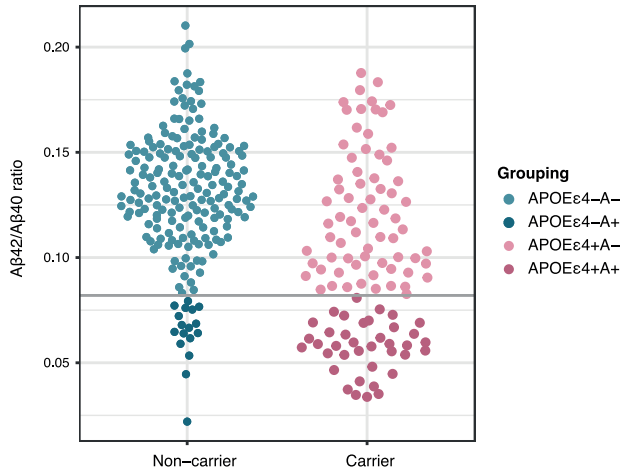

Supplement: Supplementary file 2 — Additional file 2: Supplementary Figure 1. Dichotomization of individuals into groups based on NfL concentration and APOE ε4 carrier status. [file 13195_2021_789_MOESM2_ESM.pdf]

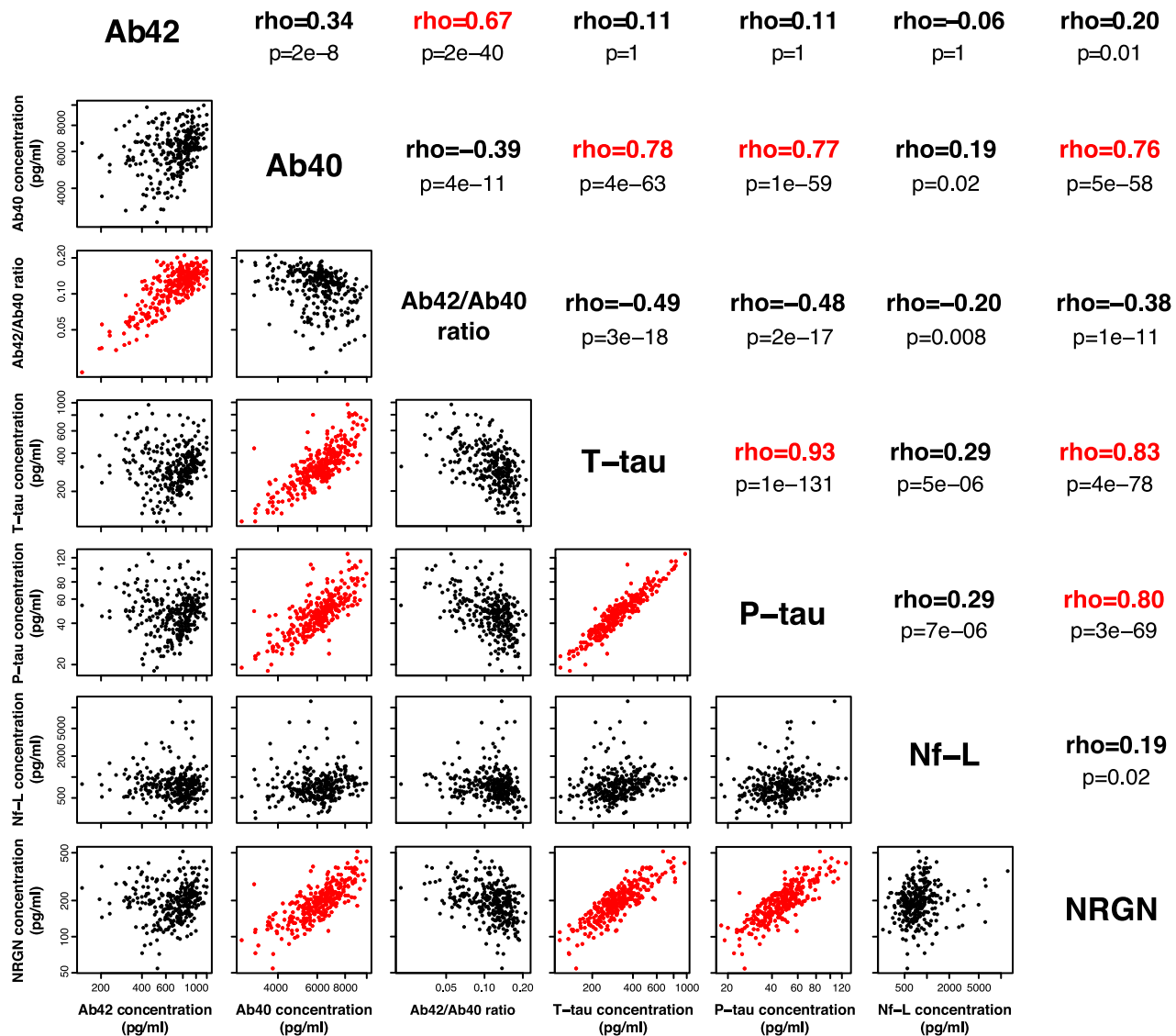

Supplement: Supplementary file 3 — Additional file 3: Supplementary Figure 2. Correlation scatterplots between suggested markers for AD, neurodegeneration or synaptic dysfunction. All correlations with a Spearman rho value> 0.6 are displayed in red. [file 13195_2021_789_MOESM3_ESM.pdf]

**A $\beta$ 42****T-tau****P-tau****A $\beta$ 42****T-tau****P-tau**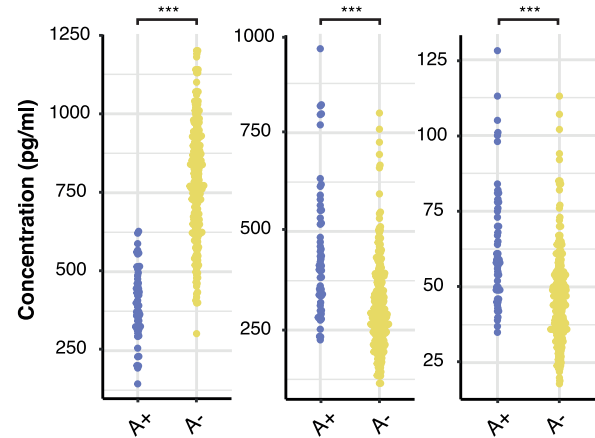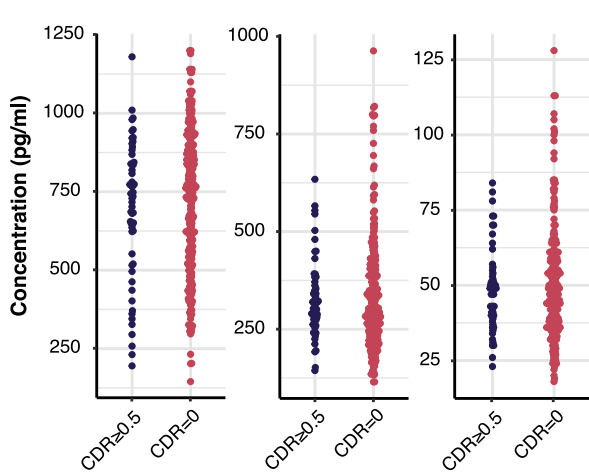

Supplement: Supplementary file 4 — Additional file 4: Supplementary Figure 3. Range of CSF markers in individuals divided by CSF Aβ42/Aβ40 ratio or CDR score. Significant differences are indicated with stars, *** p < 0.001. [file 13195_2021_789_MOESM4_ESM.pdf]

**GAP43**  
p=0.002

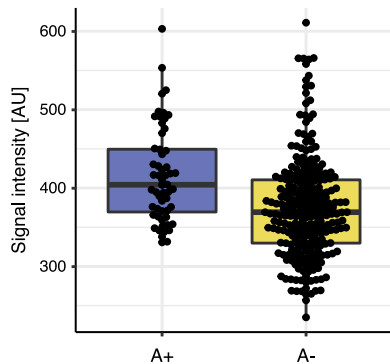

**SNCB**  
p=0.003

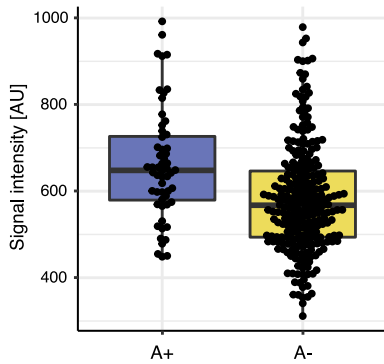

**BASP1**  
p=0.008

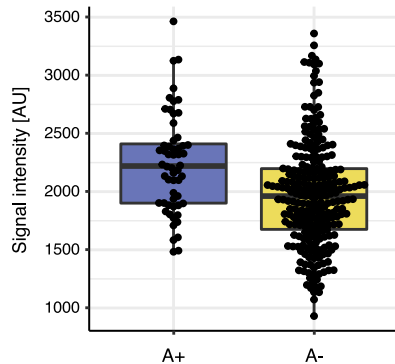

**DDAH1**  
p=0.02

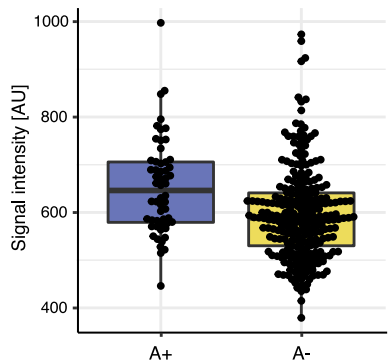

**AQP4**  
p=0.04

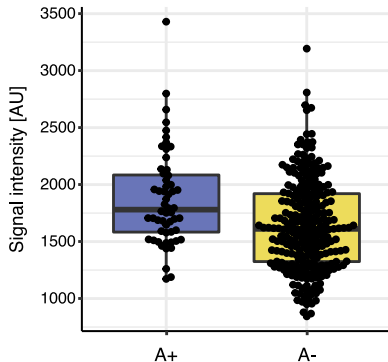

**RPH3A**  
p=0.04

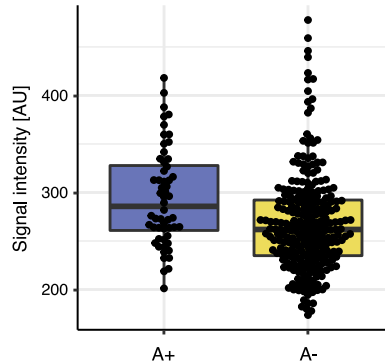

Supplement: Supplementary file 5 — Additional file 5: Supplementary Figure 4. Boxplots of proteins displaying significant differences between A+ individuals and A- individuals. [file 13195_2021_789_MOESM5_ESM.pdf]

**A**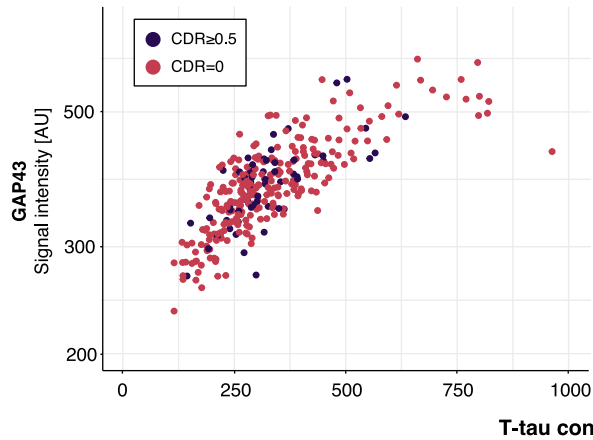**B**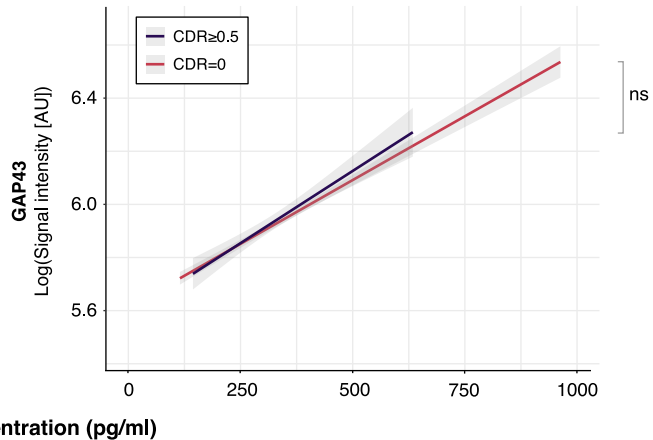

Supplement: Supplementary file 6 — Additional file 6: Supplementary Figure 5. T-tau association with GAP43 levels for individuals divided by CDR score. (A) Scatterplot of GAP43 levels and t-tau concentration. Both individuals with CDR ≥ 0.5 and CDR = 0 display significant associations of GAP43 levels with t-tau concentration (CDR ≥ 0.5: Spearman rho = 0.72; p = 3E-08; CDR = 0: Spearman rho = 0.81; p = 4E-56). (B) Linear regression revealed no significant difference between slopes of CDR ≥ 0.5 and CDR = 0 individuals for the association of GAP43 with t-tau concentration. [file 13195_2021_789_MOESM6_ESM.pdf]

### RIMS3

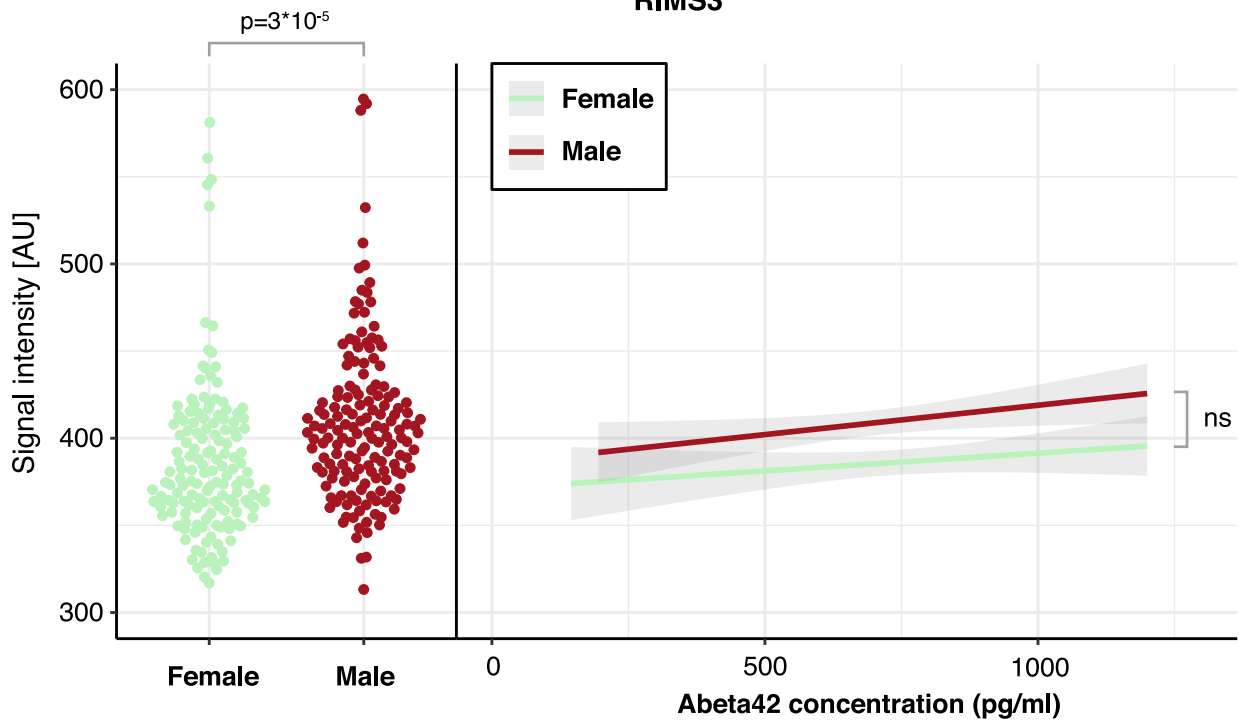

### VCAM1

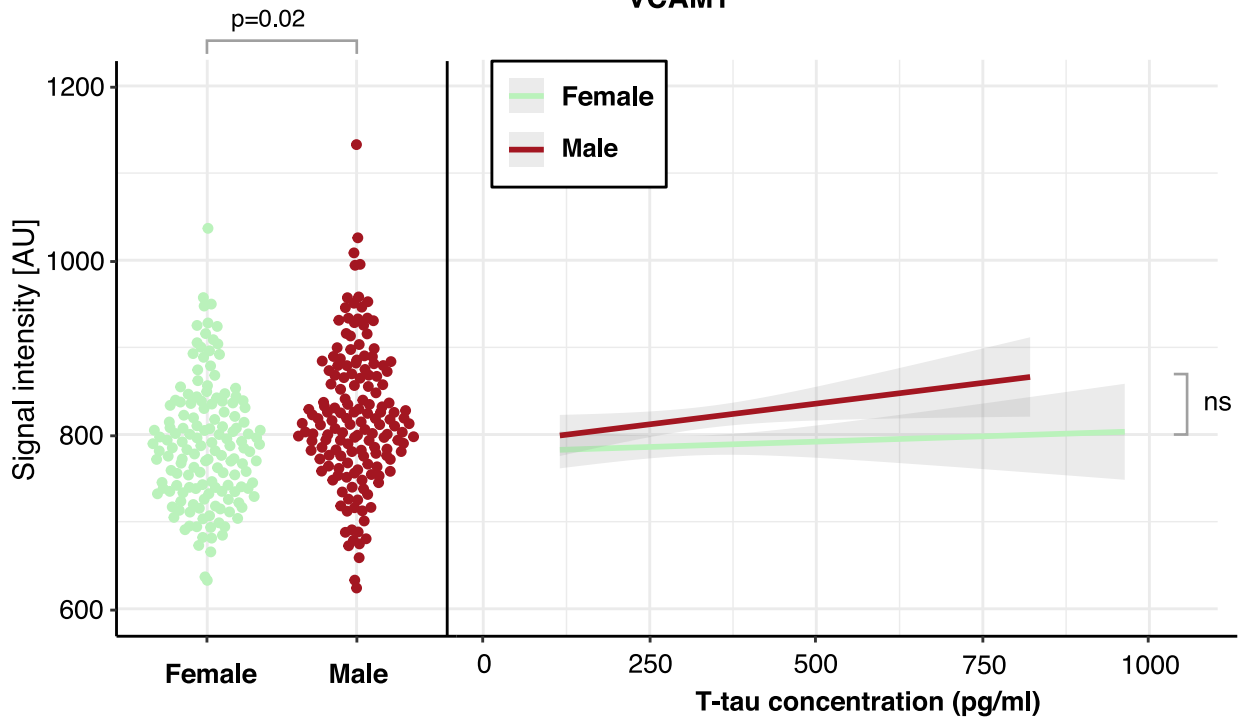

Supplement: Supplementary file 7 — Additional file 7: Supplementary Figure 6. Sex differences for RIMS3 and VCAM1. Significant differences between female and male protein levels could be identified using the Wilcoxon rank sum test. However, sex showed no significant interaction with the CSF markers. [file 13195_2021_789_MOESM7_ESM.pdf]

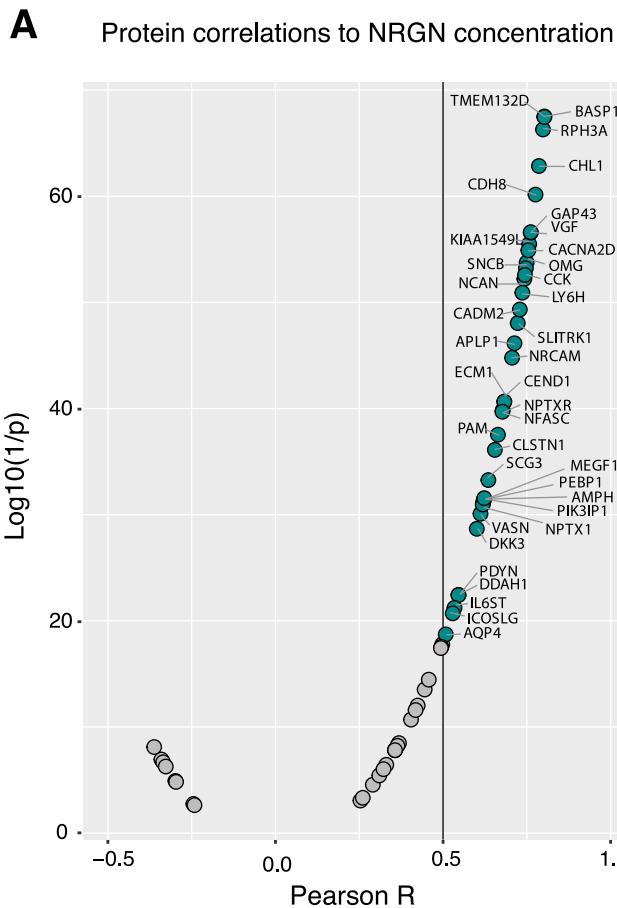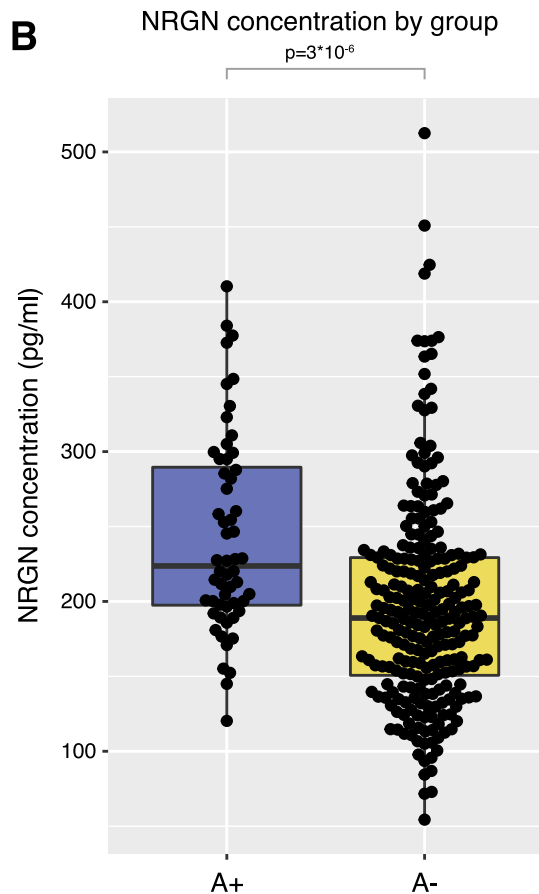

Supplement: Supplementary file 9 — Additional file 9: Supplementary Figure 8. Correlations to NRGN concentration and comparison of NRGN levels between A+ and A- individuals. (A) Visualization of Pearson R for correlations between NRGN concentration and the 104 measured proteins. Thirty-seven proteins displayed a Pearson R > 0.5 and are annotated by their HGNC ID. (B) Higher concentration of NRGN was observed in A+ individuals compared to the A- individuals. [file 13195_2021_789_MOESM9_ESM.pdf]

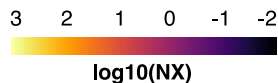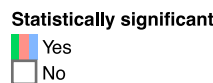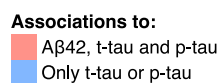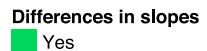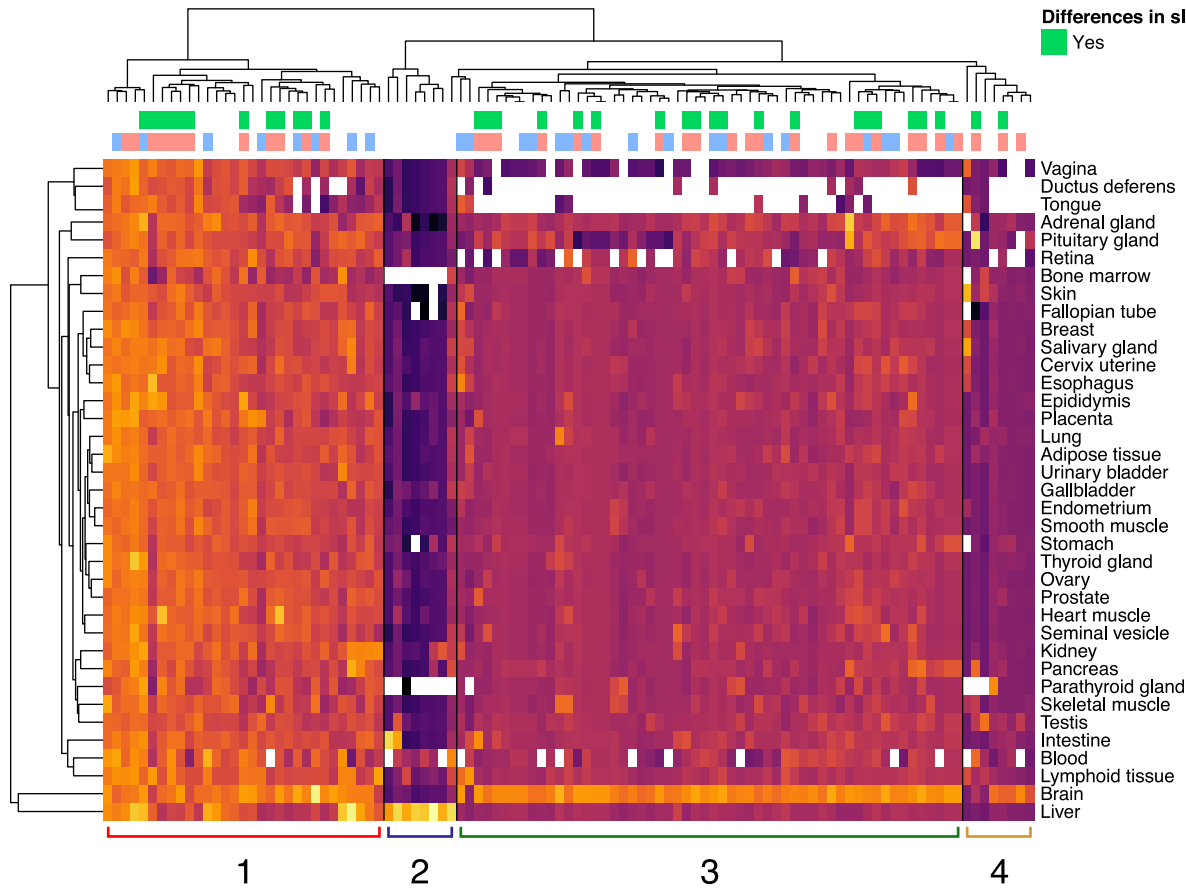

Supplement: Supplementary file 10 — Additional file 10: Supplementary Figure 9. Heatmap of tissue expression for all studied proteins across 37 human tissues. When comparing the expression profiles of the 104 analysed proteins across 37 different human tissues, the responding genes could be divided into four clusters. The first cluster showed a general expression in all tissue types (Cluster 1) and the second cluster displayed elevated expression in the liver compared to other tissues (Cluster 2). A third cluster had higher expression in the brain (Cluster 3) and the last was a mixed group with high expression in brain or other tissue types (Cluster 4). [file 13195_2021_789_MOESM10_ESM.pdf]
