# Supplementary material for: Association of CSF proteins with tau and amyloid β levels in asymptomatic 70-year-olds
Source: Alzheimers Res Ther. 2021 Mar 2;13:54. doi: 10.1186/s13195-021-00789-5 (PMC7923505; doi:10.1186/s13195-021-00789-5)
Supplement: Supplementary file 8 — Additional file 8: Supplementary Figure 7. Boxplots of proteins displaying significant differences between individuals divided by NfL concentration and CSF Aβ42/Aβ40. Upon stratification of individuals based on both CSF Aβ42/Aβ40 ratio and NfL concentration two trends in protein profiles could be identified; higher protein levels in Nf + individuals, independently of Aβ42/Aβ40 ratio and higher protein levels in the Nf + A+ group. [file 13195_2021_789_MOESM8_ESM.pdf]

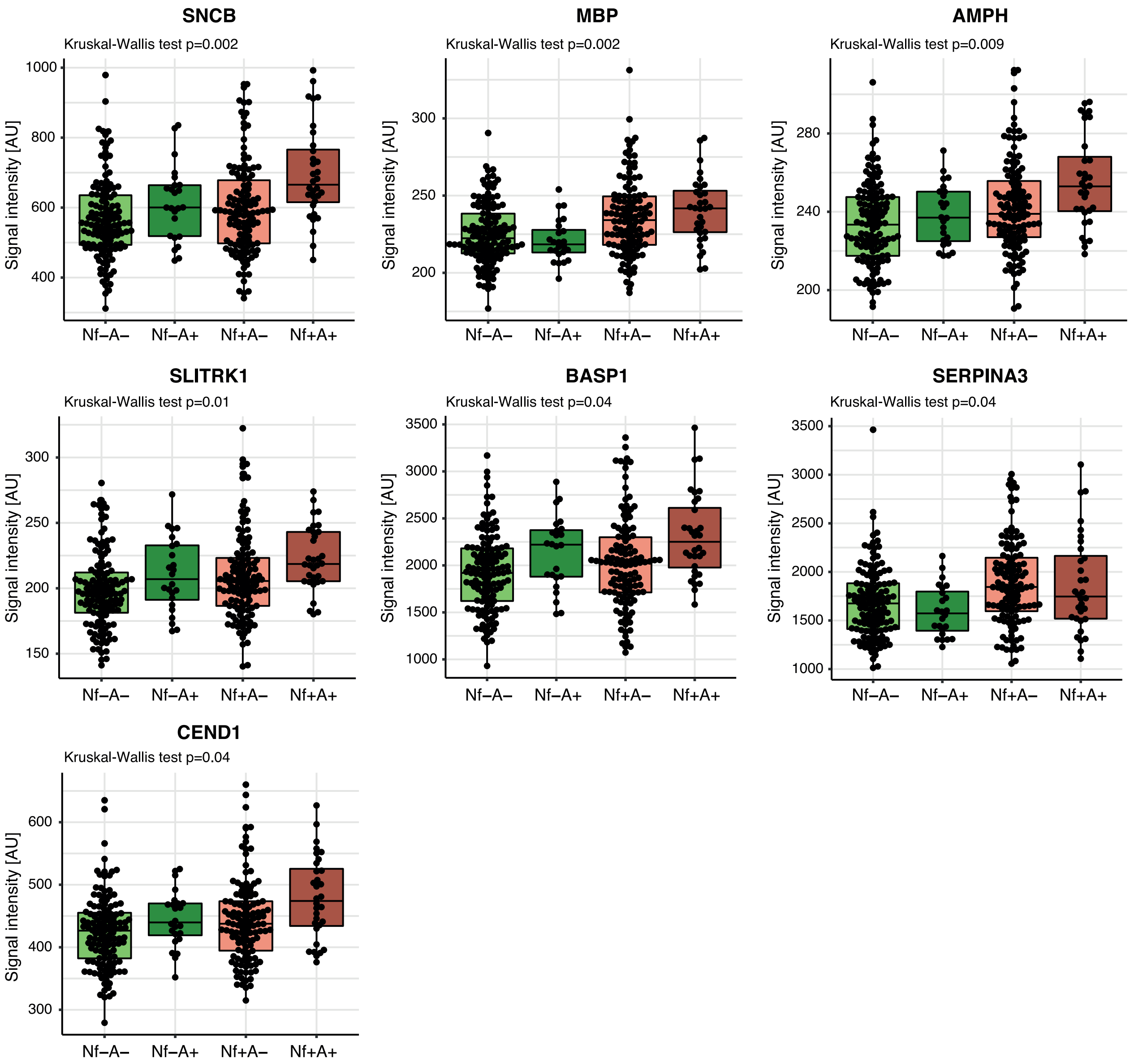
SNCB
Kruskal-Wallis test p=0.002
Signal intensity [AU]
1000
800
600
400
Nf−A−
Nf−A+
Nf+A−
Nf+A+
MBP
Kruskal-Wallis test p=0.002
Signal intensity [AU]
300
250
200
Nf−A−
Nf−A+
Nf+A−
Nf+A+
AMPH
Kruskal-Wallis test p=0.009
Signal intensity [AU]
280
240
200
Nf−A−
Nf−A+
Nf+A−
Nf+A+
SLITRK1
Kruskal-Wallis test p=0.01
Signal intensity [AU]
300
250
200
150
Nf−A−
Nf−A+
Nf+A−
Nf+A+
BASP1
Kruskal-Wallis test p=0.04
Signal intensity [AU]
3500
3000
2500
2000
1500
1000
Nf−A−
Nf−A+
Nf+A−
Nf+A+
SERPINA3
Kruskal-Wallis test p=0.04
Signal intensity [AU]
3500
3000
2500
2000
1500
1000
Nf−A−
Nf−A+
Nf+A−
Nf+A+
CEND1
Kruskal-Wallis test p=0.04
Signal intensity [AU]
600
500
400
300
Nf−A−
Nf−A+
Nf+A−
Nf+A+
